# Co-design of a Virtual Reality Cognitive Remediation Program for Depression (bWell-D) With Patient End Users and Clinicians: Qualitative Interview Study Among Patients and Clinicians

**DOI:** 10.2196/43904

**Published:** 2023-04-07

**Authors:** Maria Elena Hernandez Hernandez, Erin Michalak, Nusrat Choudhury, Mark Hewko, Ivan Torres, Mahesh Menon, Raymond W Lam, Trisha Chakrabarty

**Affiliations:** 1 University of British Columbia Faculty of Medicine Department of Psychiatry Vancouver, BC Canada; 2 National Research Council Canada Medical Devices Simulation and Digital Health Montreal, QC Canada; 3 National Research Council Canada Medical Devices Simulation and Digital Health Winnipeg, MB Canada

**Keywords:** depression, cognitive remediation, cognitive dysfunction, thematic analysis, virtual reality, VR, qualitative study, user-centered design, immersive, co-design, depressive, mental health, mental illness

## Abstract

**Background:**

Major depressive disorder (MDD) is the leading cause of global disability; however, the existing treatments do not always address cognitive dysfunction—a core feature of MDD. Immersive virtual reality (VR) has emerged as a promising modality to enhance the real-world effectiveness of cognitive remediation.

**Objective:**

This study aimed to develop the first prototype VR cognitive remediation program for MDD (“bWell-D”). This study gathered qualitative data from end users early in the design process to enhance its efficacy and feasibility in clinical settings.

**Methods:**

Semistructured end-user interviews were conducted remotely (n=15 patients and n=12 clinicians), assessing the participants’ perceptions and goals for a VR cognitive remediation program. Video samples of bWell-D were also shared to obtain feedback regarding the program. The interviews were transcribed, coded, and analyzed via thematic analysis.

**Results:**

End users showed an optimistic outlook toward VR as a treatment modality, and perceived it as a novel approach with the potential of having multiple applications. The participants expressed a need for an engaging VR treatment that included realistic and multisensorial settings and activities, as well as customizable features. Some skepticism regarding its effectiveness was also reported, especially when the real-world applications of the practiced skills were not made explicit, as well as some concerns regarding equipment accessibility. A home-based or hybrid (ie, home and clinic) treatment modality was preferred.

**Conclusions:**

Patients and clinicians considered bWell-D interesting, acceptable, and potentially feasible, and provided suggestions to enhance its real-world applicability. The inclusion of end-user feedback is encouraged when developing future VR programs for clinical purposes.

## Introduction

### The Socioeconomic Impacts of Depression

Major depressive disorder (MDD) affects approximately 5.4% of the Canadian population [1]. Most recently, the COVID-19 pandemic has increased the proportion of Canadians experiencing depression. Among people aged 25 to 44 years, the proportion of people with MDD increased from 18% in fall 2020 to 23% in spring 2021 [2]. MDD is the leading cause of disability worldwide, substantially affecting psychosocial, occupational, and academic functioning [3]. MDD costs the Canadian economy approximately >CAD $30 billion (US $22 billion) annually in reduced workplace productivity [4]. MDD also affects academic performance in young adults. University students are at higher risk of depression than general population, resulting in lower grade point averages, lower academic performance satisfaction, and higher dropout risk [5-7].

Current treatments, although effective in improving core symptoms of MDD, fall short in addressing MDD-related disability [8-10]. Symptomatic improvements are not necessarily accompanied by restoration of occupational and psychosocial functioning—patients who are diagnosed with mild or remitted depressive symptoms may still show occupational and academic disability [9,10]. Accordingly, treatment priorities have shifted to targeting functional recovery, rather than solely symptomatic remission [8,11,12]. There has been increased interest in expanding mental health services in university campuses, as well as workplace mental health programs to identify and treat employees with depression [13]. While such programs can be helpful in reducing depression symptom severity, they neither necessarily result in major improvements in workplace or academic productivity, nor in decreases in presenteeism [14-18]. Thus, depression treatment strategies specifically targeting academic and occupational recovery remains an unmet need.

### Cognitive Deficits in Depression

To enhance occupational functioning in MDD, cognition is an important intermediate target [19]. Cognitive dysfunction is often found in depressive disorders such as MDD, with deficits in memory, attention, processing speed, and executive functioning detectable in acutely symptomatic and remitted patients [20,21]. Importantly, cognitive functioning has been associated with occupational functioning in acutely depressed and recovered individuals with MDD [22-24]. However, there is a lack of effective treatments for cognitive deficits in depression. Major cognitive impairments may persist in individuals who attain symptomatic response or remission with established depression treatments [20], and antidepressant medications have inconsistent and sometimes negative effects on cognitive functioning [25].

Cognitive remediation has emerged as a promising treatment strategy for addressing cognitive deficits in psychiatric disorders [26,27]. This is a treatment method based on the repeated practice of computer or paper-and-pencil exercises that target specific cognitive skills (eg, sustained attention, verbal memory, and working memory) with the purpose of enhancing patients’ work and social functioning abilities [28]. Cognitive remediation has strong evidence for improving cognition and functioning in schizophrenia, as well as showing promise in bipolar disorder and attention-deficit/hyperactivity disorder [26,27,29]. However, these results are less consistent in depression—a recent meta-analysis of cognitive remediation in depressive disorders found moderate effects on attention and working memory, with no major effects on executive functioning or verbal memory [30]. It also remains unclear if the skills practiced during cognitive remediation in depression transfer out of the laboratory environment and result in substantial real-world functional improvements [31-33].

### Virtual Reality for the Training of Cognitive Skills

Immersive virtual reality (VR) has attributes which could enhance the delivery of cognitive remediation and has preliminary evidence for efficacy in other neurological and psychiatric disorders [34]. The immersive and interactive nature of VR facilitates greater user engagement and enjoyment compared with computer presented tasks [35,36]. VR also allows for the practice of skills in naturalistic settings, thereby improving the likelihood of skill transfer to the real world [37-39]. Randomized controlled trials have shown that VR-delivered cognitive and vocational remediation results in substantial improvements in cognition and functioning in patients with traumatic brain injury, stroke, schizophrenia, and substance use disorders, even when compared with active control conditions such as therapist administered remediation [40-43]. Additional advantages of using VR technology—which has been growing in mainstream accessibility and decreasing in expense—include its ability to reach a broader range of individuals with minimal cost. In addition, data collected from VR programs can potentially be integrated into digital health services, allowing a highly personalized intervention.

Despite the burgeoning applications of VR technologies and growing use in psychiatric populations, a sustained course of VR cognitive remediation has not been specifically evaluated in depression. As enthusiasm for using VR in clinical settings has grown, so have the questions regarding how to best design VR clinical applications to be usable and efficacious for the target population. A working group of international experts published the first consensus guidelines for best practices in the design and testing of VR clinical applications [44]. In these guidelines, incorporation of qualitative feedback from multiple end users is identified as a critical component of VR program content development. As lack of end-user involvement in development is often at the root of digital intervention failure [45], following best practices in human-centered design is important to enhance the interventions’ relevance and effectiveness. Feedback from experts and care providers can ensure that the intervention is based on solid theoretical foundations and is feasible for real-world use.

Building on the existing body of work relating to cognitive remediation in clinical populations, the National Research Council Canada and the University of British Columbia have developed the bWell Cognitive Care Platform for Depression (“bWell-D”), a prototype immersive VR cognitive remediation program for individuals with depression. Following best practices in VR clinical application design, this study collected qualitative data from clinicians and care providers regarding their perceptions and experience with VR, desired outcomes from a cognitive or functional remediation program, and perceived barriers to use of VR cognitive remediation in clinical settings. Later, participants provided their thoughts and opinions specifically regarding the bWell-D program. This information was used to guide program and protocol refinements to bWell-D to boost clinical efficacy and feasibility.

## Methods

### Ethics Approval

This research was reviewed and approved by the Behavioural Research Ethics Board of the University of British Columbia (Behavioural Research Ethics Board numbers H21-00028 and H20-00746). It was also approved by the Research Ethics Board of the National Research Council Canada (NRC 2020-122).

### Participants

#### Patients

A sample of 15 patients who self-reported a diagnosis of depression were recruited to participate in this study. Recruitment was carried out on the web through the Vancouver Coastal Health Research Institute and REACH BC websites, as well as on a classified advertisements website. Interested participants were screened to ensure they met the eligibility criteria:

1. Aged 18 to 65 years

2. Self-reported diagnosis of previous or current major depressive episodes

3. Self-reported subjective cognitive or functional deficits at baseline, as indicated by a battery of self-report questionnaires. All participants were included in the analysis, regardless of their scores in the clinical measures.

4. Engaged in full-time employment (currently or in the past), with self-reported ongoing functional deficits resulting from depressive symptoms, or off work or on reduced hours with depressive symptoms reported as the primary reason.

5. Sufficient proficiency in English to complete the questionnaires and interview

Patients who were eligible and completed the entire process were compensated for their participation with CAD $20 (US $14.55).

#### Clinicians

A convenience sample of 12 clinicians participated, with recruitment occurring through the Vancouver Coastal Health Research Institute and REACH BC websites, as well as physical posters displayed on-site at relevant health units. Clinicians were also contacted through the researchers’ professional contacts. Interested clinicians were provided a letter of invitation and confirmed their interest in participating via email. Clinicians must have been allied health professionals (eg, psychiatrists, psychologists, family physicians, occupational therapists, or nurses) with experience working with populations with depression, and experience with cognitive or functional remediation in psychiatric populations. Clinicians were not offered a monetary compensation.

### Procedure

This study was conducted remotely through videoconferencing. Participants attended an approximately 1-hour Zoom interview, in which they were first explained the purposes of the study, and oral informed consent were obtained. Participants then completed a web-based Qualtrics survey asking for demographic data. In addition, patients completed a series of psychiatric and psychological questionnaires (see the *Measures* section), whereas clinicians answered questions related to their clinical practice. After completing the questionnaires, a semistructured interview was conducted, in which participants were asked regarding their perceptions and experiences with VR, their desired outcomes from a workplace cognitive or functional remediation program (bWell-D), and perceived barriers to use this type of treatment in a clinical setting. Participants were then shown previously recorded video samples of the bWell-D tasks and were asked to provide their thoughts on them, their potential relevance to daily life, and suggestions on how to improve the tasks to enhance engagement and real-world applicability (see Multimedia Appendix 1 for the interview scripts).

### The bWell-D Program

#### Overview

“bWell-D” is a prototype of immersive VR cognitive remediation program for depression, developed by a collaboration between the University of British Columbia and the National Research Council Canada. The foundational platform, bWell, was developed within a network of researchers and clinicians across Canada, features configurable exercises and design as a broadly applicable toolkit targeting general aspects of cognition commonly affected across many disorders [46]. Preliminary acceptability studies in healthy individuals indicate that the platform is enjoyable, engaging, and well tolerated [35,47].

**Figure 1:**
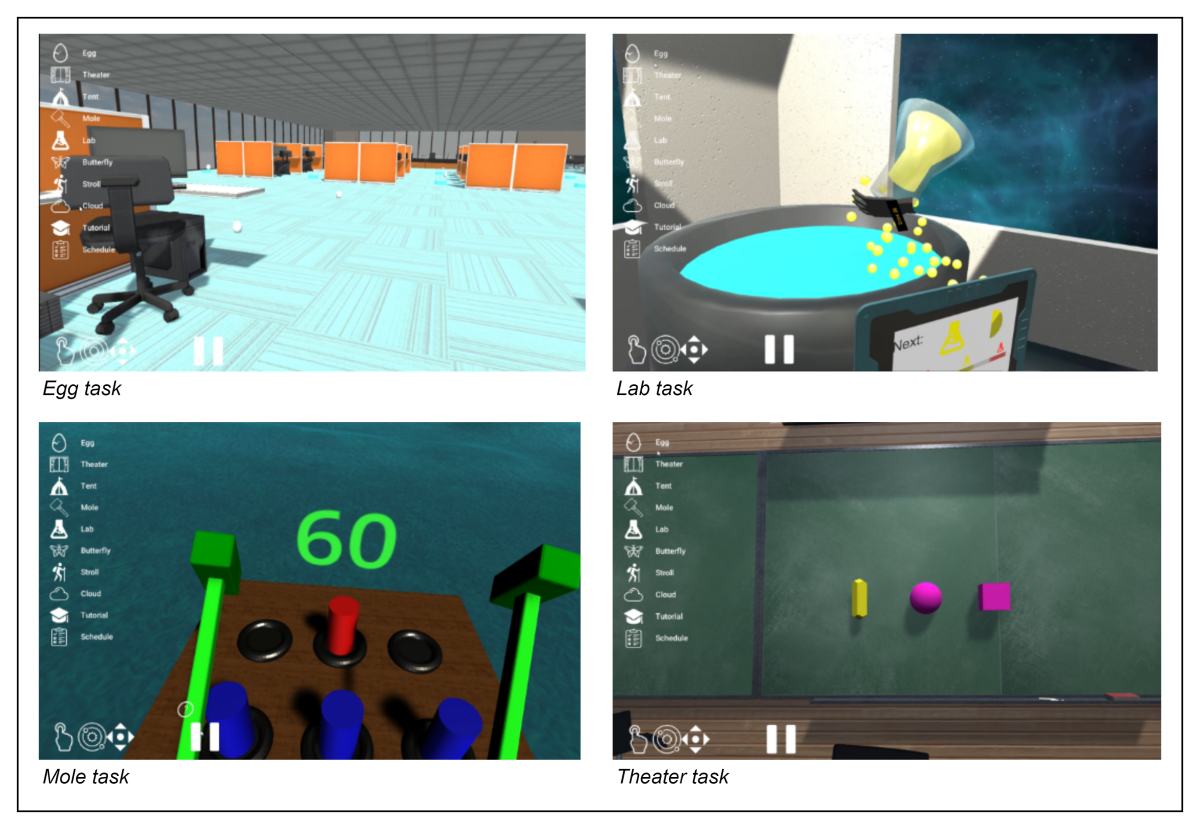
Screenshots of the bWell-D tasks.

bWell-D is a customized version of bWell, targeting cognitive and real-world challenges germane to MDD depression [20,21,46]. Two bWell tasks were modified to include real-world environments, that is, the (1) office and (2) classroom. It additionally uses previously identified common components of successful cognitive remediation in psychiatric populations. These include (1) “errorless learning,” wherein participants are provided support in learning all task components, with supports gradually removed as skills and confidence increase and (2) “adaptivity,” in which tasks become increasingly complex to match with the participant’s competence [31]. bWell-D involves 4 different tasks (Figure 1) that aim to train cognitive skills that are commonly affected in MDD. Participants can interact with the virtual environment through a headset and 2 hand controllers.

#### Egg (Attention)

In this task, the patient is asked to look around in an office environment and look for eggs. Once locating the egg with their sight, they are required to fix their gaze on the egg until it hatches.

#### Lab (Multitasking)

Participants must complete 2 recipes simultaneously by pouring multicolored flasks into 2 mixing bowls. The recipes will appear in 2 tablets placed nearby, and participants will have to go back and forth between recipes.

#### Mole (Reaction Time and Response Inhibition)

Following similar rules to the whack-a-mole game, participants hold a hammer in each hand and hit the cylinders that pop out of a table. The cylinders are multicolored, and the colors of the hammers also change over time. Participants are asked to hit the cylinder that matches the hammer’s color.

#### Theater (Visual Working Memory)

The participants are shown a sequence of shapes that will be hidden after a set viewing time. A pool of objects will then be presented to the participant, from which they must choose the shapes that were initially shown to them and recreate the original sequence.

The bWell-D tasks operate in three modes: (1) tutorial, (2) assessment, and (3) training. Tutorial has the aim of familiarizing the patient with the virtual environment and showing them the actions they must perform in the tasks. Assessment mode has the purpose of identifying the patients’ individual needs by assessing their task performance with a fixed difficulty level. In the training mode, the level of difficulty is adaptable based on the patients’ performance. Real-time feedback is provided to the patient, showing them level or score changes and successes and errors.

### Measures

Along with the demographic questions included in the Qualtrics questionnaire, patients also responded to the following psychiatric and psychological measures:

#### Lam Employment Absence and Productivity Scale

The Lam Employment Absence Productivity Scale [48] is a work functioning and productivity scale to be used specifically with patients with depression. It is a 10-item scale, in which the first 3 questions are open-ended and ask regarding the patients’ type of work, the number of working hours scheduled in the past 2 weeks, and the number of working hours missed in those 2 weeks. The following items are rated on a 5-point Likert scale (“None of the time” to “All the time”), scored as 0 to 4. Work impairment rating can range between “None to minimal” to “Very severe.”

#### Generalized Anxiety Disorder–7 Items

The Generalized Anxiety Disorder–7 items [49] is a short measure of anxiety. Participants are asked how often they have been bothered by their anxiety symptoms during the past 2 weeks. The measure follows a 5-point Likert format, ranging from “not at all” (0) to “nearly every day” (4).

#### Sheehan Disability Scale

The Sheehan Disability Scale [50] is a short measure of disability and functional impairment. The first 3 questions are in a Likert scale format, inquiring regarding the severity of cognitive symptoms affecting the patients’ work and social and family life from “not at all” (0) to “extremely” (10). The last 2 open-ended questions ask regarding the patients’ missed days at work, and regarding the days when the patient felt underproductive.

#### Perceived Deficits Questionnaire–Depression

The Perceived Deficits Questionnaire–Depression [51] is a 20-item questionnaire that assesses subjective cognitive dysfunction in people with depression in the past 7 days. It follows a 5-point Likert scale, with response options ranging from “Never” (0) to “Almost always” (4).

#### Beck Depression Inventory

The Beck Depression Inventory [52] is a 21-question, self-report Likert scale that measures the severity of depression symptoms. Responses range from 0 to 4, in which a higher number indicates a more pronounced symptom severity.

#### Cognitive Failures Questionnaire

The Cognitive Failures Questionnaire [53] is a measure of self-reported failures in perception, memory, and motor function over the past 6 months. It is a Likert scale consisting of 25 items ranging from “Never” (0) to “Very often” (4).

### Qualitative Data Analysis

Interviews were recorded and transcribed verbatim. The transcriptions were made with Otter.ai, a web-based service that uses artificial intelligence to transcribe audio recordings. The transcriptions were reviewed and corrected by hand after being processed by Otter.ai. The initial 5 interviews (3 clinician interviews and 2 patient interviews) were coded by the first author to identify an emerging thematic framework [54]. Coding was conducted separately for the clinician and patient groups. However, members of the research team (EM, TC, and MEHH) discussed and identified emerging patterns in the data and determined that the coding framework between the 2 groups was similar; therefore, it was decided to analyze the 2 groups together. Subsequent transcripts were coanalyzed and discussed based on the initial thematic framework. Thus, the data analysis began with the delivery of the first interview and proceeded concurrently and iteratively. The NVivo (QSR International) software was used to aid with the coding and thematic analysis process.

## Results

### Participant Characteristics

#### Patients

Participants were aged between 21 and 63 years, with a median age of 31 (mean 34.2, SD 11.5) years. Most participants self-identified as women (n=10), with 3 as men and 2 as nonbinary. The most common diagnosis was MDD (n=8), and 1 patient was diagnosed with dysthymia, 1 with bipolar disorder, 4 of them reported being diagnosed with depression but did not remember the specific diagnosis, and 1 did not have a formal depression diagnosis, but manifested having all the clinical symptoms for it (this was later confirmed at the screening stage). Patients with a formal diagnosis reported being diagnosed between 3 and 16 years ago. At the time of the interview, none of the patient participants were undertaking any psychological treatment, however, 4 of them were taking medication to treat their depression symptoms.

Patients’ clinical scores are provided in Table 1. Overall, participants surpassed the threshold for a clinical diagnosis in all measures. All participants reported having their work or academic performance affected by their depression symptoms, and all of them have had to take some time off work or school, ranging from a few sporadic days, to several years. Participants stated working only approximately 80% of their scheduled work hours, some of them having to quit work completely because of the severity of their depression symptoms. Furthermore, 9 patients were either currently employed or studying, while 6 of them were unemployed and identified depression as one of the main causes for their unemployment. From the participants who were employed at the time of the interview, 4 had administrative jobs, 3 had professional jobs, and 2 worked in sales.

**Table 1:** Patients’ clinical measures.

Measures	Values, n	Values, median (range)	Severity^a^
LEAPS^b,^^c^	9	15 (4-29)	Moderate
GAD-7^d^	15	19 (9-27)	Severe
SDS^e^	15	22 (6-30)	Markedly impaired
PDQ-D^f^	15	46 (7-69)	Risk range
BDP^g^	15	25 (8-47)	Moderate depression
CFQ^h^	15	62 (15-79)	Above average cognitive complaints

^a^On the basis of median scores.

^b^The LEAPS is only for participants who are currently engaged in paid employment, hence the reduced sample who responded to this specific measure.

^c^LEAPS: Lam Employment Absence and Productivity Scale.

^d^GAD-7: Generalized Anxiety Disorder–7 items.

^e^SDS: Sheehan Disability Scale.

^f^PDQ-D: Perceived Deficits Questionnaire–Depression.

^g^BDP: Beck Depression Inventory.

^h^CFQ: Cognitive Failures Questionnaire.

According to the open-ended questions in the Lam Employment Absence Productivity Scale, employed participants had between 50 and 80 scheduled work hours in the previous 2 weeks, and although most of them worked their full shifts, 2 participants only worked between 15 and 20 hours. Four participants were currently working reduced hours, and they have been in this work modality between 2 months and 1 year. The responses to the open-ended questions of the Sheehan Disability Scale indicated that employed participants had lost between 1 and 20 days of work in the past month because of their depression symptoms. Even when attending work or school, participants reported that depression affected their performance in the last month, ranging from 30% of the days to 50% of the days. One participant reported feeling these effects daily.

#### Clinicians

Participants self-identified as 5 women and 7 men. There were 7 psychiatrists, 2 occupational therapists, 1 psychologist, 1 clinical counselor, and 1 neuropsychologist. They had varied years of experience as mental health practitioners, ranging from 2 to 50 years, with a median of 17.5 years. Five clinicians reported having <5 years of experience in clinical practice, while 6 of them reported having >20. One clinician reported having between 5 and 10 years of experience.

Most of the clinicians worked in an outpatient setting (n=10), either at hospital clinics or private practice. Only 1 clinician worked at an inpatient psychiatric hospital, and another one worked at a community health center. In total, 7 clinicians provided a consultation and assessment and short-term follow-up model of care, 3 of them offered long-term follow-up, and 2 of them did single consultation and assessment with no follow-up. Furthermore, 4 clinicians offered primarily pharmacological and medication treatment modality, 3 of them offered primarily psychotherapy, 3 of them a combination of medication and psychotherapy, and 2 focused primarily in functional rehabilitation. Clinicians who provided psychotherapy offered behavioral treatments, such as cognitive behavioral therapy, dialectical behavior therapy, and acceptance and commitment therapy.

A total of 7 clinicians estimated <50% of their patients had a depressive disorder, while the remaining 5 estimated that more of the 50% of their patients had depression. Five clinicians had experience with cognitive or functional remediation, and they had between 0.5 and 5 years of experience with this type of treatment. Most clinicians (n=9) had been working remotely because the start of the COVID-19 pandemic, mostly relying on videocalls, phone calls, and (less commonly) computer tasks with their patients.

### Thematic Analysis

#### Overview

Participants held mostly positive opinions regarding bWell-D, considering it an entertaining and safe treatment modality. Five themes were identified during the thematic analysis, specifically participants’ previous knowledge regarding VR technology, its potential for clinical uses, a desire for realistic settings and situations, the entertaining features of VR, and the need for an inclusive treatment. A description of each theme and representative quotes are presented below.

#### Familiarity and Optimism: Openness to Trying VR for Mental Health Symptoms

Both patients and clinicians had at least some degree of familiarity with VR. Patients were familiar with VR mostly from videogames and movies, while clinicians were also aware of its use for clinical purposes, especially for anxiety-related conditions, psychosis, and brain injury. When participants did not have direct experience with VR, they mentioned hearing good things regarding it. Participants with little or no exposure to the technology spoke of their curiosity regarding trying it.

I mean, I’m curious to see if it could, in any way, help my own mental health issues. And, I don’t know, it’s something I haven’t tried yet. And I’m open to trying things that might improve my wellness.

Patient 003; woman; 43 years

I don’t really know too much about it. But I’m very open to learning more. I guess, like I don’t know enough to say whether I see potential or not, so I’m eager to learn more.

Clinician 005; woman; occupational therapist

Participants with a higher exposure to VR, and the ones who enjoyed videogames, were the ones who showed higher enthusiasm regarding this technology.

#### VR as a Potential but Underexplored Treatment Option for Cognitive and Functional Deficits in Depression

Although both patients and clinicians often considered mood symptoms as the most salient symptom in depression, they acknowledged that cognitive symptoms are also affected. They described cognitive issues as particularly harmful for work life, because they have a direct effect on job performance, which can in turn exacerbate feelings of shame and stigma:

It was just a really hard time. I just, I couldn’t concentrate, well, I still have a hard time concentrating. Just my, my work ethics went down. I don’t know, everything became really hard. I missed a few days here and there, like I felt, but at the same time, like even though I was told by the doctors like, ‘No, you need to take time off,’ I kept going because I didn’t want to be judged behind my back. But if anything that made it worse [...] But I know that my, my work has gone down, like, and I have a really hard time concentrating.

Patient 001; woman; 21 years

Several patients considered that training their cognitive skills through VR can help them not only for work purposes but also with their day-to-day life:

I go into a room to get band aids, I forget what I’m in there for, literally my house is not that big. And I go back in the living room wondering what the hell I was looking for. So it, I feel like it would help with work and with my personal life, which is just a double bonus.

Patient 003; woman; 43 years

They were confident that their cognitive abilities can be re-established after experiencing depression. When asked regarding the potential applications of VR for depression, 2 clinicians and half of the patient sample struggled to see how this technology could be used for this condition:

I’m aware of the promise of virtual reality treatments for anxiety disorders and things like that. I would be, I don’t see the application to depression or bipolar disorder. So you know, in that end, those are the two disorders I treat the most. So therefore, I’m not particularly looking at this. And I’m surprised to hear about a virtual reality treatment for depression

Clinician 009; man; psychiatrist

Participants considered VR a safe alternative to conventional psychiatric treatments, and most patients were willing to try it because of its perceived lack of serious side effects. Participants believed that more effective treatments for mood disorders are needed, and therefore, VR can be a promising alternative.

I don’t always enjoy the medication approach, like the side effects can be quite frustrating at times. So I think something without, like, a less medicalized approach could be really helpful. And you could avoid some of the negative side effects.

Patient 014; woman; 21 years

I think we need all the treatments we can get for mood disorders. So I’m curious.

Clinician 009; man; psychiatrist

Participants believed that VR is a novel approach to treat depression, and because it is becoming increasingly popular for clinical purposes, they would like to explore this treatment modality further:

I think will become more common in the future. So I think it might be like a good way to look into it and learn about it before it like the, as is, you know, gaining more popularity.

Clinician 005; woman; occupational therapist

A few participants, however, considered this “newness” a limitation to start using VR, because it is not standard practice in most health care institutions:

I practice in a large university hospital type setting. The university hospital always is incredibly bureaucratic, and never wants anything that’s innovative or different. They only want things that are standard. So anytime you try to introduce something new, it’s very challenging. So I would say that it would be unlikely that even if this treatment were approved tomorrow by Health Canada or the FDA, I would say it’s very unlikely that our system would use it for anytime in the next few years.

Clinician 009; man; psychiatrist

Despite the general enthusiasm regarding using VR and cognitive remediation, both patients and clinicians had some further reservations, and mentioned needing more evidence before undertaking a treatment of this nature. Most clinicians mentioned that, although this type of treatment has shown promising results in some conditions, they would like to have more evidence for its specific use in depression. They would like to obtain this information from methodologically sound research studies:

If there is going to be evidence for that, for the type of patients that I’ve seen, and the patients are willing to participate? So yeah, for sure [...] It’s not part of the kind of the regular practice, or part of the standard guidelines. But once the evidence is there, for sure.

Clinician 012; man; psychiatrist

Furthermore, approximately half of the patient sample mentioned that the VR format might not have any particular advantages over other modalities of cognitive remediation delivery:

I think the only like, pushback that might be received is like, what is the difference between doing this and playing like, a task related like, video game, I guess? Like, I think that there’s an, there’s maybe an opportunity for, like, people who are depressed to be like, “Well, if I if I’m basically just going to a lab to play a game, what’s the difference between me doing that, and me just like downloading a game on my phone where you’re being asked to, like, do memory parts?”

Patient 008; woman; 30 years

#### Desire for Ecologic Validity

Several patients mentioned that they would like to understand exactly how the tasks practiced in bWell-D work, so they do not feel like they are “just playing a game.” They also mentioned needing an explanation regarding how to extrapolate the trained skills to their real-life world:

My honest opinion on the games, they all seem like kid games. They all seem very simple and straightforward, but simple animation. And I find like, I might think it’s just a silly game, but I think once I start actually playing them, I would realize how it’s helping.

Patient 007; woman; 28 years

I think I would need the therapist to, like, fully explain what this was training and helping with, because, like, the one thing that triggered me when I was in a depressive state was that, like, I knew I was smart, but then like, when I was in a depression, I wasn’t functioning, that, like, as normal, and then I would feel really, really dumb. And like, seeing this kind of activity makes me feel like, you know, it gives a reaction like, I’m not a child, like, why am I putting shapes together?

Patient 10; woman; 27 years

Most participants mentioned that VR offers the possibility of immersing themselves in settings and situations that resemble real life in a highly accurate manner:

It’s very interesting in the way that all new technology is very interesting. And it’s remarkable, you know, the sense of presence when you’re in the VR environment. It, you know, unless you’ve experienced it, it’s hard to imagine, you know, it’s very much like being in the real world.

Clinician 011; man; psychiatrist

Most patients mentioned they would like the virtual environments to look like their own work or school setting, as well as including objects (eg, office supplies or school furniture) and situations (eg, conversations with colleagues, following instructions given by their boss or teacher) that emulate real life (eg, interactions with humans and distractions). They believed that these naturalistic aspects can enhance skill extrapolation:

I think that it would also require some effort relevant to their daily activities, or have some translational component.

Clinician 004; man; psychiatrist

I think if I work in an office, it would, it would be better because I’d be able to take what I’m doing within that virtual reality, and apply it more to the environment that I’m hoping to improve my concentration in. But because it doesn’t look like where I work it’s difficult to connect the two.

Patient 007; woman; 27 years

Participants considered VR as “the second-best thing to real life,” because it allows an immersion in highly realistic environments. Participants desired to obtain this realism through several ways, (1) by having virtual settings that emulated patients’ own real-life environments, (2) by including distractors in the VR world that would occur in real life, and (to a lesser extent) (3) by having realistic-looking graphics. Patients and clinicians also stated that VR environments allow for the practice of skills and exercises in a more controlled and less intimidating setting:

If I were able, was able to get better at that, and because it’s a low stress environment in the game, maybe having it, having that experience in a low stress situation would help me in my more stressful situations.

Patient 014; woman; 21 years

It’s like the traditional thing when you, when you’re nervous about speaking in front of a crowd is that, you like, you practice your speech in the mirror. It could be something similar to that but in a VR setting, with there being more of a...I don’t quite know how to articulate it, exactly.

Patient 005; nonbinary; 25 years

A few patient participants mentioned that VR can allow them to focus on only training their skills without getting sidetracked. However, they emphasized the need for improving their cognitive symptoms overall, rather than just improving their performance in the virtual tasks:

I’m skeptical because of the literature that’s behind this, because there’s a lot of evidence saying that you only get better at the game instead of the skill itself, or the cognitive aspect that you’re focusing on itself.

Patient 001; woman; 21 years

Three clinicians mentioned the importance of ensuring that the skills practiced in programs like bWell-D are actually training what they intend to train, and that the tasks are representative of the actual cognitive processes that patients must perform at their work or day-to-day life:

If there’s evidence that depressed people have trouble on that sort of task, it makes sense. If you’re pulling it out of thin air, because they have trouble on an attempt and inhibition task that’s not like that one, then you better rethink how close that task mimics the process you’re interested in.

Clinician 011; man; psychiatrist

Approximately a quarter of the participants were concerned regarding the long-term effectiveness of the treatment. To address this, a patient suggested having a lengthier VR cognitive remediation treatment, whereas a clinician suggested adding booster sessions. Several participants expressed a desire to obtain treatment progress data through a reliable instrument. Although a few of participants mentioned wanting improved graphics in bWell-D (more realistic looking), others mentioned that this is not too important if the content of the tasks is well designed, and the objects are clear to see. Most participants mentioned that any possible glitches and malfunctions should be minimal or nonexistent for the treatment to be effective and enjoyable.

#### A Fun and Engaging Treatment: The Advantages of a Digital Environment

Most participants mentioned that the gamified experience of VR adds fun, engaging, and immersive elements to the treatment. A need for multisensory stimuli (ie, tasks that incorporated auditory and visual components) was mentioned by most of the participants and was considered key for the immersion and involvement in the virtual tasks. Participants also stressed the importance of having a difficulty progression in the tasks, to enhance the challenging aspect of the treatment (without it turning so difficult that it becomes discouraging). A clinician mentioned how this difficulty progression can provide a sense of accomplishment for patients:

It definitely gives you kind of that drive, while at the same time being complicated and interesting enough to keep your attention wanting to keep going [...] – I found that one was always a really fun task, a difficult task. Not difficult, but it required enough energy without being overwhelming.

Patient 015; woman; 33 years

Most clinicians and patients stressed the importance of having a simple, user-friendly VR interface that included some training period and clear instructions to practice the tasks. A few clinicians considered important to assess patients’ satisfaction with the program session to session.

Several participants, especially clinicians, found the versatility of VR to be beneficial for the treatment. They noted that the flexibility provided by VR allows a wide variety of virtual settings, going beyond what is achievable in real life:

I think you can help us create a very realistic environment to do some therapeutic work in and potentially make some progress that we haven’t been able to outside.

Clinician 007; woman; psychologist

A few participants considered a treatment, such as bWell-D less emotionally draining treatment modality than medications or talking therapy:

When I was watching it, it occurred to me it’s kind of nice to have these videos not relating to real life experiences, and things that are supposed to be kind of fun, because it doesn’t feel like you’re doing work, even though you are. And that is, at least for me, isn’t as emotionally or cognitively draining.

Patient 015; woman; 33 years

Seven participants (both patients and clinicians) mentioned that the game-like setting of VR also allows for progress tracking (eg, score and level changes and improvements), either from the beginning to the end of a session, or from session to session.

#### One Size Does Not Fit All: The Need for Inclusivity and Customizability

Most patients and clinicians mentioned that they would like to have some agency in the manner the VR treatment is conducted. One of the most recurring comments among patients and clinicians was a preference for the patient to have the treatment individually at home, either partially or completely. Participants believe this modality provides a sense of comfort and control:

It seems like a nice way to do it yourself. Like, in your own time in, in quiet, in a quiet environment and not have to go anywhere.

Patient 002; woman; 36 years

I think it would be helpful for people who can’t get there, like remotely. Like, there are days when I can’t like, get out of bed or like, get overwhelmed by taking the bus, and like, that might be super helpful to be able to join in like that.

Patient 10; woman; 27 years

However, a few patients and clinicians believed a treatment of this nature would be better performed at a clinic. In particular, patients with limited space, or with suboptimal conditions to carry out the treatment at home, would like to have the option to do it at a clinic if necessary:

If I had to use it at my own home, that would be almost impossible with three kids and, I think, life.

Patient 012; womana; 42 years

Participants mentioned wanting a person involved in the treatment even if they choose to do it remotely. Several patients stated that the involved person should be a well-trained facilitator who they trusted. Most of the patients and clinicians mentioned they would like to engage in a VR treatment when mood symptoms and other potential comorbidities are under control, and therefore had enough motivation to undertake the treatment:

It depends on the severity, so I think it could be quite hard for some individuals to sustain, you know, attention or memory, if they’re having more severe symptoms. But I think as a symptom, sort of subside or set, you know, it’s more mild to moderate, we might be able to focus a bit more and pay attention to it a bit better.

Clinician 007; woman; psychologist

I think like when you are depressed, like, it’d be really hard [...] if there’s any sort of resistance in that process. I think like, a really anxious person or really depressed person would be like, ‘Nope, I can’t deal with it. It’s not working. I’m done with it. Not going to use it today’ sort of mentality.

Patient 008; woman; 30 years

In fact, several patients and a few clinicians mentioned the usefulness of including an activity in bWell-D that specifically targeted mood. After looking at the task video samples, 4 patients also mentioned that some tasks could be more useful than others (eg, patient not currently struggling with memory issues or wanting to focus more on multitasking). Therefore, they would like to tailor the treatment according to their own cognitive struggles and needs. A clinician also commented that, although the office or school-like VR settings could be useful to emulate naturalistic settings, these settings can trigger emotional responses from some people:

For people who are not working, many people with depression are on short or long term disability from work, I was just wondering if seeing the office environment might trigger something.

Clinician 010; woman; psychologist

Despite the overall positive perceptions regarding bWell-D, approximately half of the clinician sample and one patient stated they would like to do a VR treatment in parallel with other conventional depression treatments (eg, medications or psychotherapy):

You target that symptom, but then you work with the clinician long term to, to use this as sort of one of the other interventions that you’re using, and I would see this not being a single intervention being used. I see this being used in combination.

Patient 001; woman, 21 years

Another of the most mentioned topics among patients and clinicians was how age could be an important factor when undertaking a VR cognitive remediation treatment. In particular, participants believed that the more technologically savvy individuals (often the younger generations) would like the treatment better. In turn, participants believed that older patients might need more coaching and adjustment time to become used to bWell-D. Several participants also believed that people prone to sensorial issues might dislike a VR treatment, because it can make them feel physically ill:

The other problem with all of this is that people go into their depression with some of them, depression of course worsens all of this, it can worsen this, but some people have pre-existing audio processing difficulties or visual processing difficulties either genetically or by head injury or a stroke or medical injuries, etc..

Clinician 002; man; psychiatrist

Participants also mentioned that the treatment should be accommodating to people from different backgrounds and with different needs, such as people with color blindness or visual impairments, people whose English is their second language, and people with limited mobility. One of the most common concerns was the actual access to the bWell-D program and the necessary equipment (eg, headset, controllers, or computer). Most participants acknowledged that these technological devices, although they are becoming cheaper with time, may still be inaccessible for people with more limited economical resources:

They [VR sets] cost a little bit of money, and most people with mental illness don’t have any money. So yeah, just having access to it, having access to a headset

Patient 004; man; 49 years

The job that I work, I don’t have coverage yet, and when I do get coverage, I don’t know how much it is. And maybe, I’m not sure if the city offers it, like if it’s readily available. So I guess cost and availability would be the biggest challenges for that.

Patient 007; woman; 28 years

However, a clinician mentioned that, if the treatment shows to be effective for patients, then every effort should be made to open access to it.

### Changes Made to bWell-D After Qualitative Feedback

Following the guidelines proposed by Birckhead et al [44], the changes suggested by end users to improve the bWell-D program were discussed among the research team, translated into representative software functionalities during team brainstorming sessions, and were given priority for implementation based on (1) how frequently these changes were suggested by end users and (2) whether these changes were feasible for development in a VR environment. The main areas of improvement suggested by both clinicians and patients were based on the thematic analysis, and mainly centered in (1) taking greater advantage of the potential of VR to create more multisensorial tasks; (2) the inclusion of more ecologically valid VR elements (to reflect more realistic VR environments), as well as having bridging exercises to relate the skills trained in bWell-D to real-world situations; and (3) providing sufficient challenge for fun and engaging tasks. In addition, end users suggested having a user-friendly interface and tutorials to have an opportunity to practice the tasks, which they considered especially important for an at-home treatment. Moreover, participants recommended to include an additional VR activity that specifically targets their mood. Participants believed that these modifications could make bWell-D more interesting, engaging and useful to improve cognitive deficits. The implemented functionalities are described in Multimedia Appendix 2, while Multimedia Appendix 3 provides a more in-depth description of these changes, and Multimedia Appendix 4 shows some visual examples.

Although efforts were made to cover as many of the suggested changes as possible to bWell-D, there were some areas that the research team determined to address with alternative strategies. In particular, one of the suggestions made by several participants was the addition of more VR environments that better emulated patients’ real-life settings, such as their own workplace or school. However, the research team concluded that the addition of these VR environments would be too demanding in terms of resources required for software development. Instead, it was decided that such need would be addressed trough bridging exercises, which are clinician-led discussions that help cognitive remediation participants to apply what is trained in the tasks to everyday life [55].

As part of the iterative process in the design of bWell-D, the changes made to bWell-D were later shown to a subset of 4 patient participants, once more as prerecorded video samples. After looking at the updated version of the program, participants were provided with additional information regarding bWell-D and were asked regarding their general opinions regarding the implemented changes (see Multimedia Appendix 5 for the reinterview script). All the participants reacted positively to the changes, and considered them an improvement to the initial version of the program.

## Discussion

### Principal Findings

The main aim of this study was to explore the opinions of patients and clinicians regarding the use of VR for the treatment of depression-related cognitive disfunction. bWell-D, a VR treatment specifically designed for treating cognitive symptoms of depression, was used as the main VR treatment example for the participants. The results indicate that VR interventions for treating depression and its cognitive symptoms are generally accepted by both patients and clinicians. In the particular case of bWell-D, they considered it an innovative and fun alternative for the treatment of mental health issues. Some suggestions were made for the improvement of bWell-D, such as including multisensorial tasks, cues, and distractors, a progression in the difficulty of the tasks, realistic virtual settings and activities, having a user-friendly interface, including tutorials and an opportunity to practice the tasks before initiating the treatment, and adding a task or VR activity that targets patients’ emotional state.

Although the general perceptions regarding VR for clinical purposes and bWell-D were mostly positive, participants also expressed some reservations. In particular, clinicians needed more evidence before implementing a treatment of this nature in their own clinical practice. Although there is a growing body of evidence supporting cognitive remediation [56] and VR in health care settings [57], structural obstacles within health institutions, as well as health professionals’ own personal preferences (ie, an inclination for more traditional therapeutic approaches), can prevent them from being open to use strategies or technologies that deviate from their usual practice. This phenomenon has been previously described as resistance to change [58], and it is one of the main reasons why novel approaches in the treatment of several conditions remain underused [59].

Similarly, patients mentioned wanting a thorough explanation regarding how the skills trained in programs such as bWell-D can extrapolate to the real world. To this end, psychoeducation has been identified as a key component in the treatment of depression [60], which could address the utility of training cognitive skills as a means to achieve recovery. This psychoeducation component has been found useful by patients in previous studies, such as the study by Lindner et al [61]. It is worth mentioning that 3 of the younger participants considered the VR tasks too infantile or simple to relieve cognitive symptoms of depression, which could be an indication of a generational preference regarding the type of tasks offered through bWell-D. As a result, the inclusion of realistic paraphernalia in the VR environments, as well as the increasing difficulty of the tasks, can potentially address these concerns.

Patients also commented regarding the need of a customizable VR treatment in which they could decide over how, when, and where the treatment was conducted. There was a strong preference for doing the treatment individually at home, because this was considered the most convenient modality for patients. Although considering patients’ opinions within any psychiatric or psychological intervention is essential, clinicians should be mindful regarding not losing key components of the intervention when accommodating patients’ preferences. By doing so, clinicians might be drifting away from best practice, preventing patients of receiving the best treatment available [62].

There was an overall positive perception toward VR interventions for depression and other mental health conditions, and toward bWell-D itself. Participants indicated that they would use this type of treatment if available, and this acceptance was mainly motivated by the fact that a VR intervention was perceived as a treatment with no adverse long-lasting consequences or side effects. Participants believed that, in the worst-case scenario, the treatment may not improve their cognitive skills but would not worsen them, which was an advantage when compared with other more conventional treatments, such as medication. This relative safety provided by VR treatments can be highly beneficial by end users, given that, besides the economical investment, there are little to no risks associated with this treatment. However, the accessibility concerns manifested by end users should be a consideration for implementation and future directions in the bWell-D development process, given that currently there are no clear plans regarding how this treatment will be made available for clinicians and patients.

This study shares some specific findings with previous literature. In the study by Kramer et al [63], clinicians also indicated a need for more empirical evidence to support the use of VR as an assessment or therapeutic tool, and they also expressed a concern regarding how the use of VR faces challenges regarding its adoption and assimilation. In the study by Thompson et al [64], participants were also worried regarding technological difficulties, mentioning how they might not have access to the necessary equipment to undertake the treatment, and how including a VR intervention in their routine might interfere with patients’ schedules. The lack of perceived adverse effects was also highlighted in the study by Nason et al [65], in which patients experienced only minimal motion sickness. As in our own study, Krebs et al [66] found that relatable humanoid avatars are important in virtual environments, given that they promote the suspension of disbelief, which is the key difference between a gamified intervention and a more traditional one. The results of our research join the large body of studies in which VR interventions for mental health issues are perceived positively by end users [67-69]. However, to the best of our knowledge, this is the first study that analyses qualitatively the use of cognitive remediation for cognitive symptoms of depression.

### Limitations and Strengths

This research had some limitations. Further insights might have been obtained from participants if they have had hands-on experience with bWell-D, instead of watching prerecorded samples. While it was initially intended that participants would trial bWell-D before completing the qualitative interview, COVID-19 pandemic restrictions prohibited on-site testing during the data collection period, necessitating the change to the virtual format used here. Related to these COVID-19 pandemic restrictions, patient participants were recruited through various web-based sources and self-reported their depression symptoms. Future studies might benefit from in-person assessments in which a health care professional administers the clinical questionnaires. In addition, most of the clinician sample did not have practical experience with cognitive remediation, clinicians with direct experience could have clearer expectations of a VR cognitive remediation treatment and may have therefore provided more extensive feedback. The sample included both people who were still working or in school but had depression-related impairments, as well as people who were off work—these 2 groups might conceivably have different concerns and needs from cognitive remediation. Finally, the clinician sample was formed mostly by psychiatrists working in outpatient settings—a more diverse clinician sample might have provided broader perspectives regarding the use of cognitive remediation and VR for depression. In terms of strengths, this is, to our knowledge, the first study that focused on obtaining end-user feedback regarding a VR intervention for cognitive symptoms of depression with a qualitative approach. We were able to obtain rich and extensive feedback from a varied sample that included patients from a wide range of ages, backgrounds, and genders, which offers an inclusive and comprehensive perspective toward the use of VR and cognitive remediation. Finally, reinterviewing the participants reaffirmed the relevance of the changes made to the program and enhanced the user-centered design, which will continue to be central in the iterative process of bWell-D development until its application in clinical populations.

### Conclusions

Virtual interventions for the improvement of cognitive symptoms in depression are generally acceptable and potentially feasible for patients and clinicians. Although VR and its use for the treatment of health-related conditions is relatively new, participants were familiar with it and open to try it, given its perceived safety. They were curious regarding a treatment of this nature and saw potential in its implementation. Participants considered it to be important to have realistic virtual settings, tasks, and cues to enhance the extrapolability of the skills trained in the VR program. The virtual format of this intervention was perceived as an advantage, and participants believed it offered a versatile and fun alternative to conventional depression treatments. Patients’ own needs and preferences should be considered when implementing a VR treatment for psychiatric conditions, and all efforts should be made to make it more accessible and convenient for end users.

## Appendix

Multimedia Appendix 1 Interview scripts.

Multimedia Appendix 2 Modifications made to bWell-D tasks after end-user feedback - Overview.

Multimedia Appendix 3 Changes made to bWell-D - Description.

Multimedia Appendix 4 Examples of modifications made to bWell-D after end-user feedback - Screenshots.

Multimedia Appendix 5 Re-interview script.

## Abbreviations

MDD: major depressive disorder
VR: virtual reality
