# Supplementary material for: Co-design of a Virtual Reality Cognitive Remediation Program for Depression (bWell-D) With Patient End Users and Clinicians: Qualitative Interview Study Among Patients and Clinicians
Source: JMIR Serious Games. 2023 Apr 7;11:e43904. doi: 10.2196/43904 (PMC10131700; doi:10.2196/43904)
Supplement: Multimedia Appendix 4 [file games_v11i1e43904_app4.pdf]

Egg

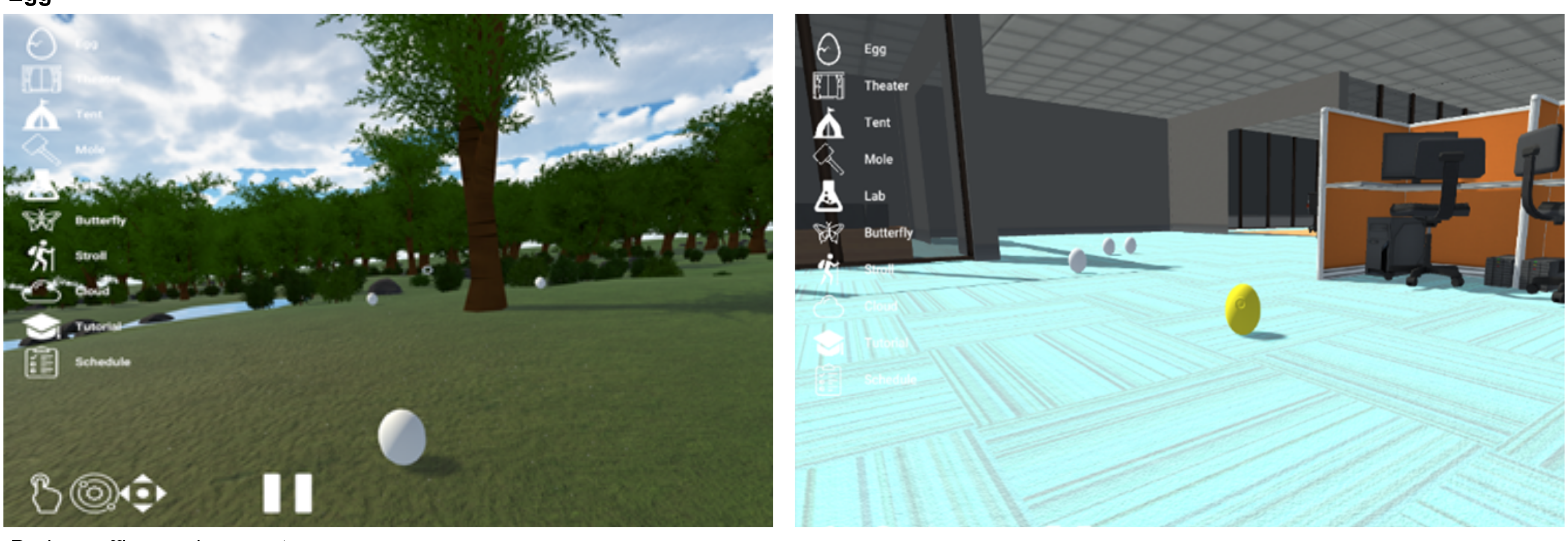

Park vs office environment

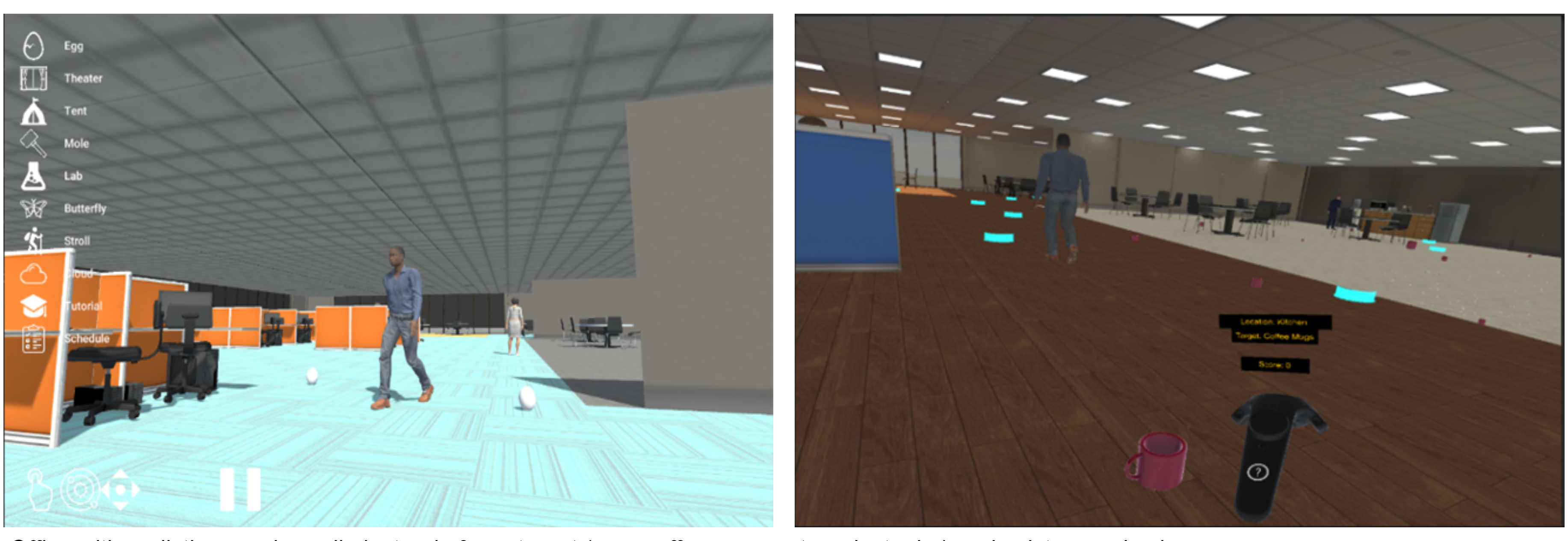

Office with realistic paraphernalia instead of egg target (e.g., coffee mug, notepad, stapler) and wrist menu in view

Lab

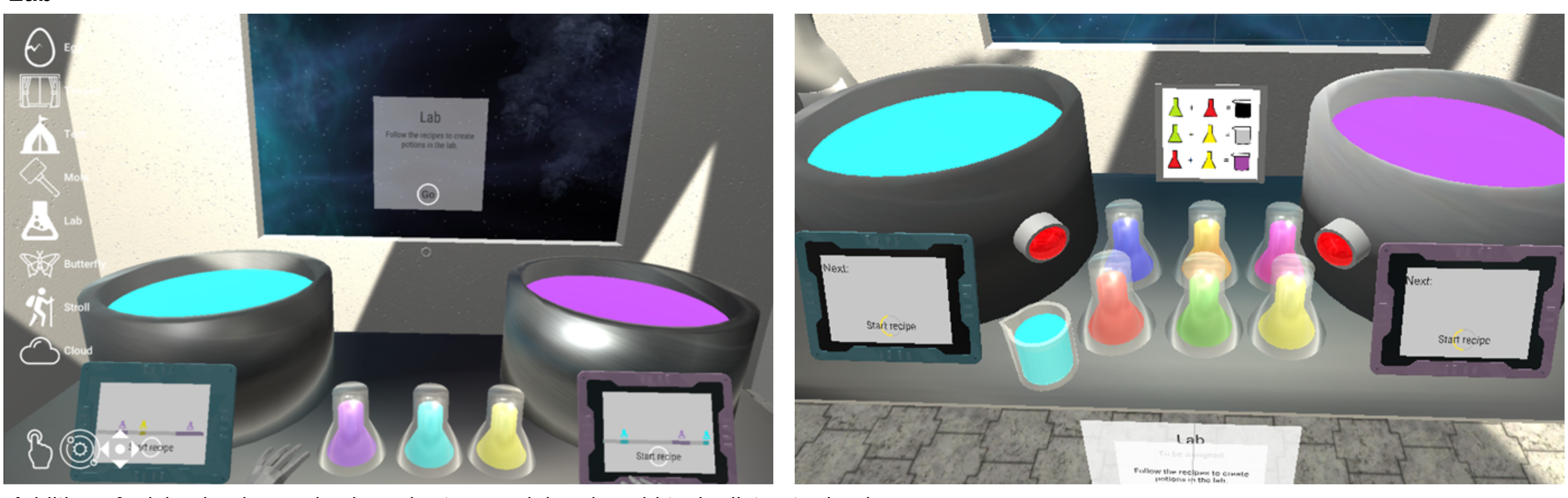

Addition of mixing beaker and colour chart as a mini real-world task, distractor beakers

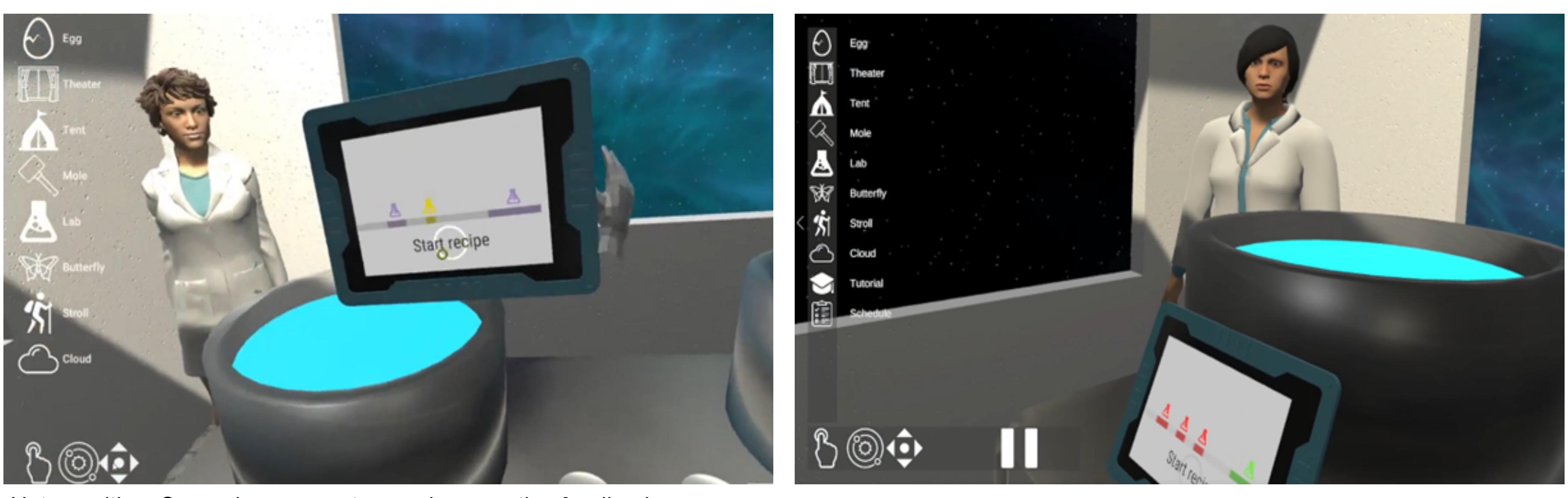

Hot cognition: Supervisor present, can give negative feedback

Mole

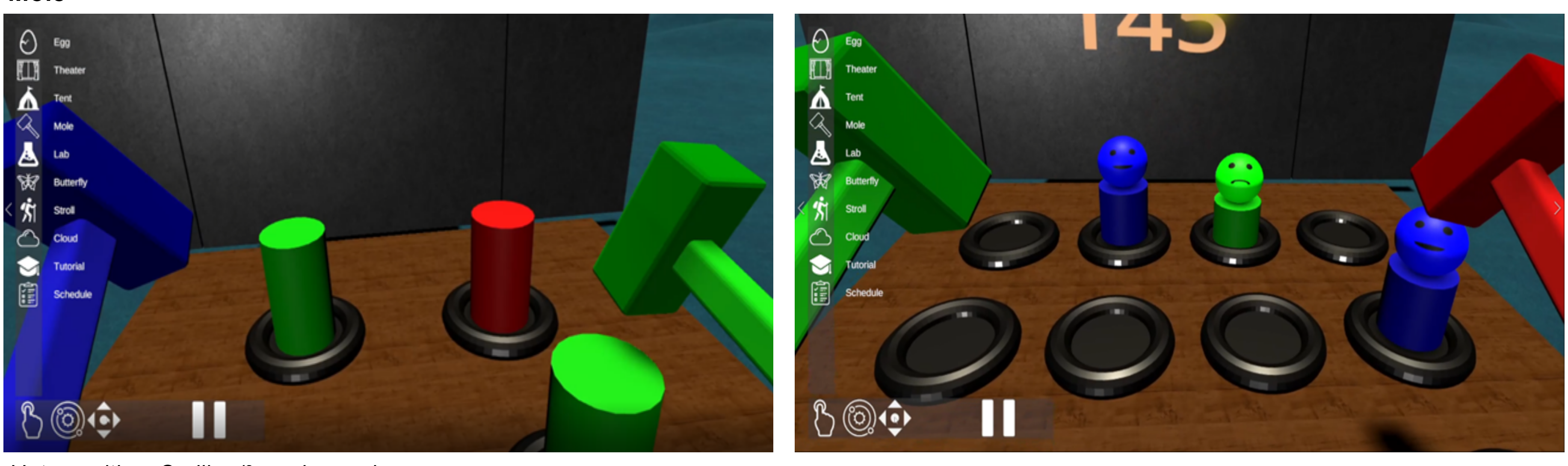

Hot cognition: Smiling/frowning moles

Theater

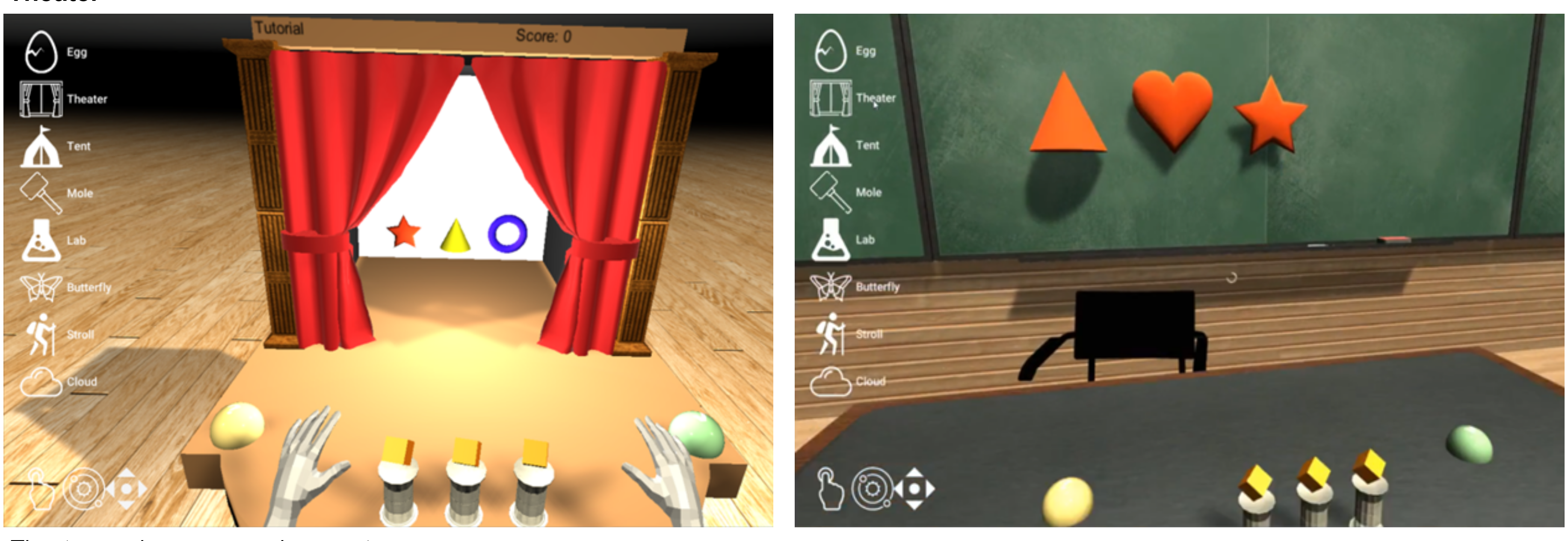

Theater vs classroom environment

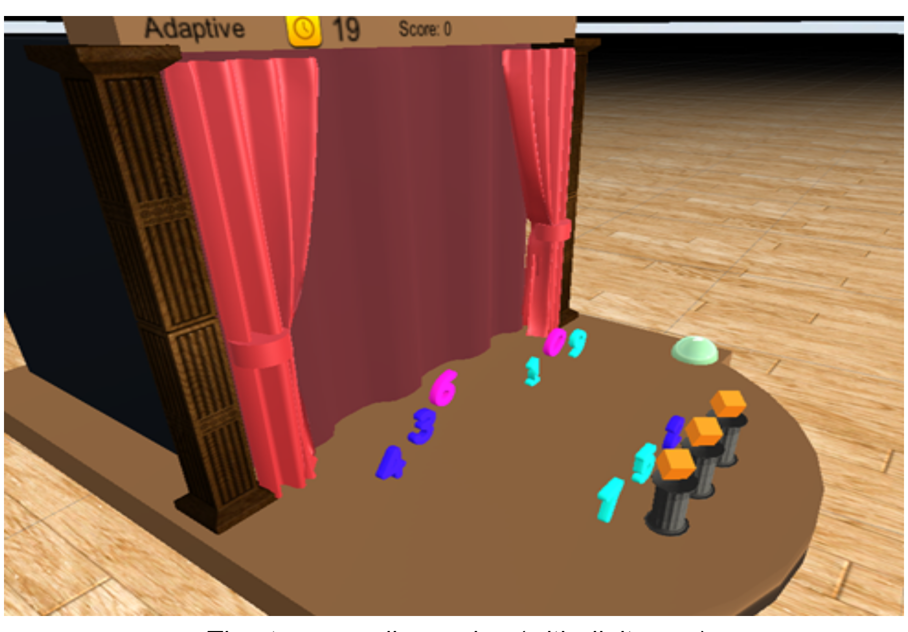

Theater as audio version (with digit span)

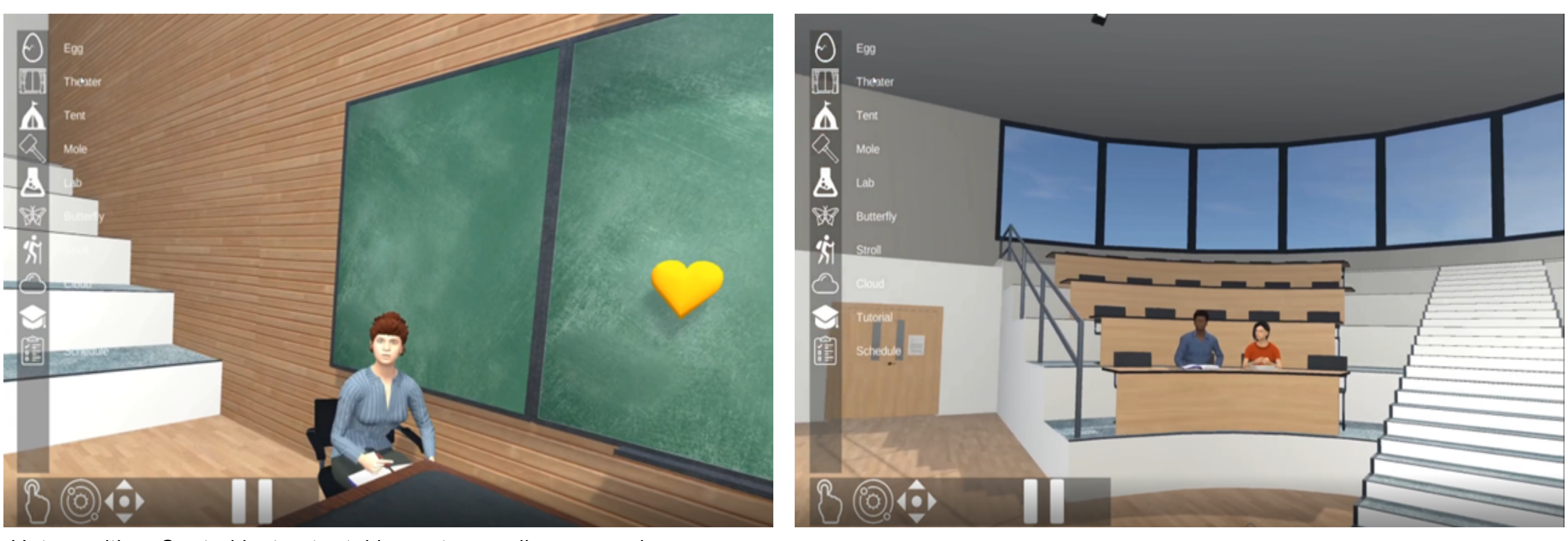

Hot cognition: Seated instructor taking notes, audience members

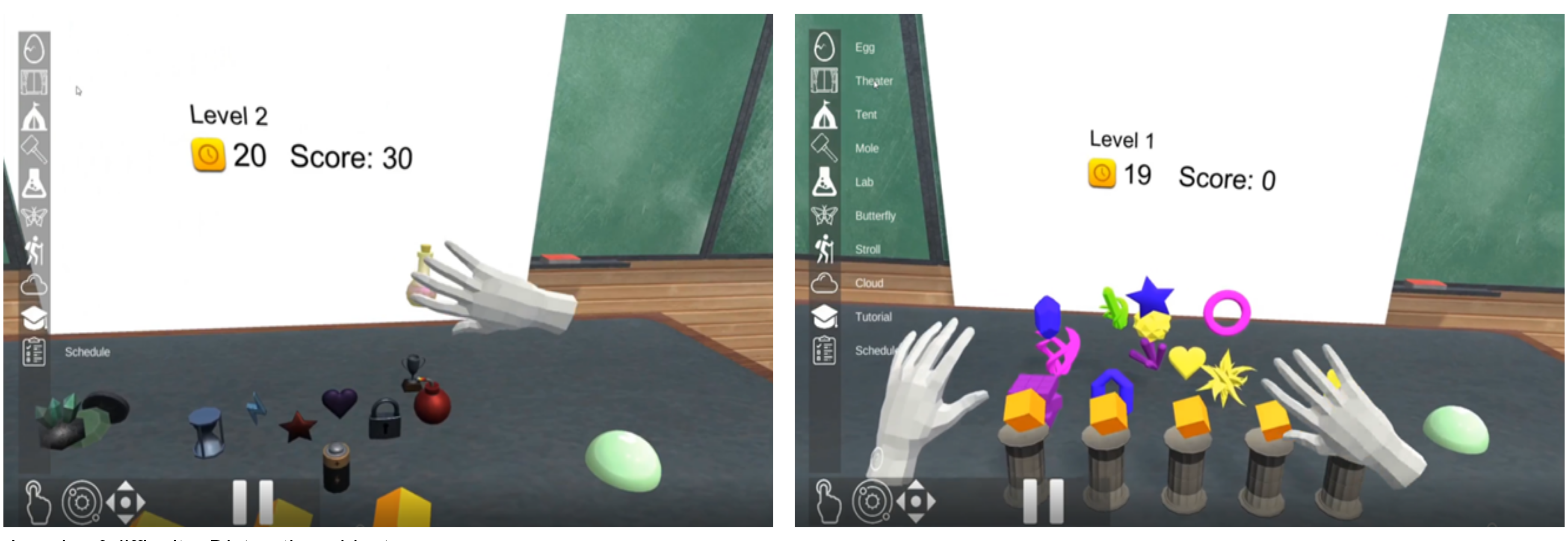

Levels of difficulty: Distracting objects

Tent

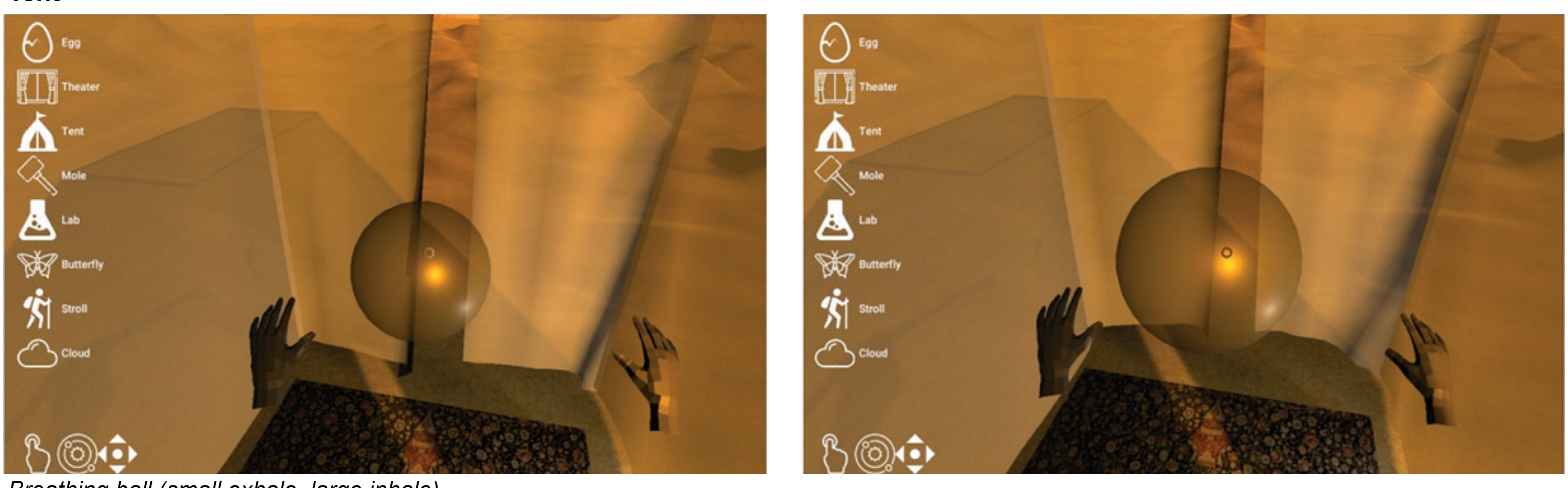

Breathing ball (small exhale, large inhale)

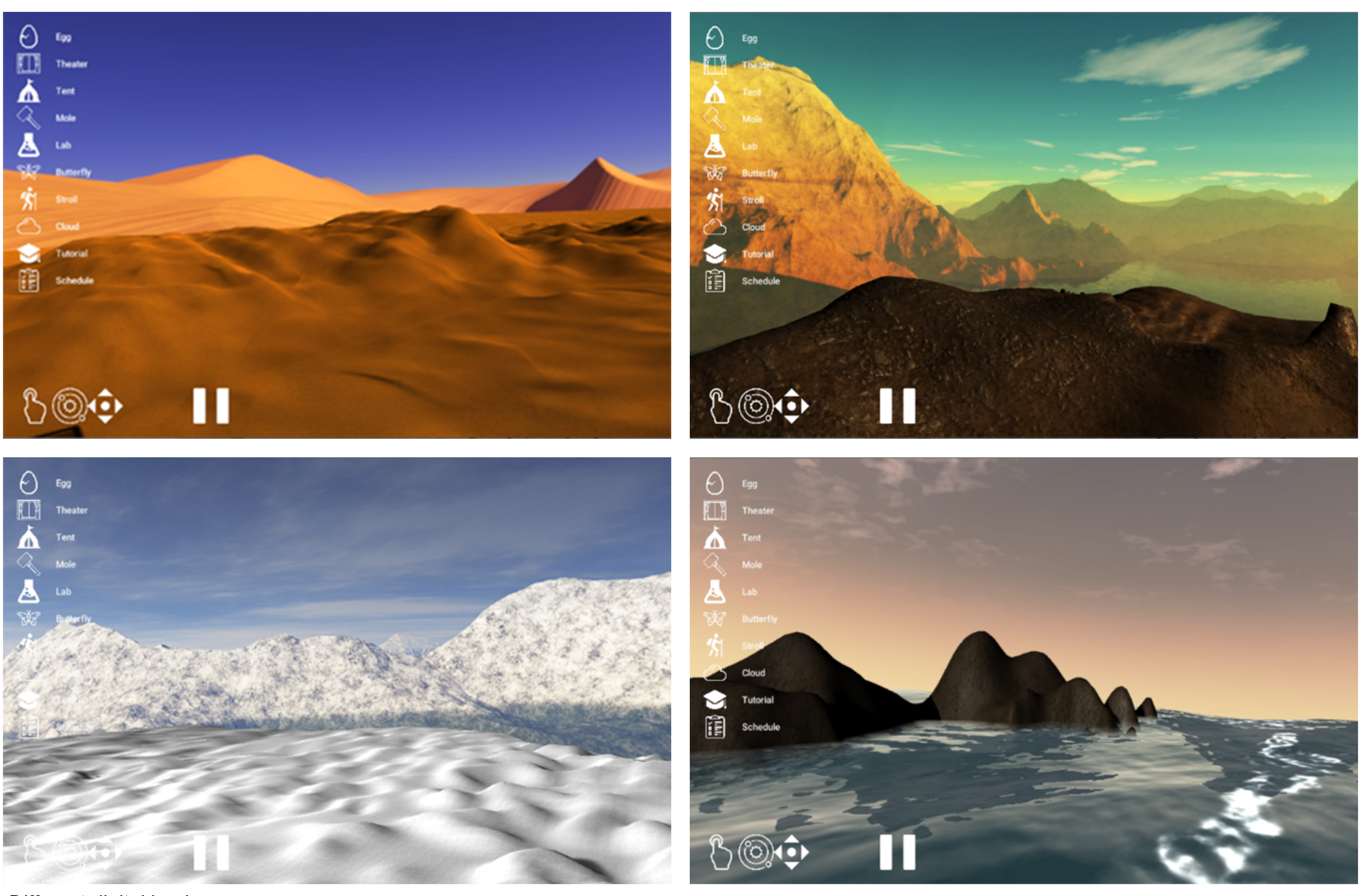

Different digital landscapes
