# Supplementary material for: Co-design of a Virtual Reality Cognitive Remediation Program for Depression (bWell-D) With Patient End Users and Clinicians: Qualitative Interview Study Among Patients and Clinicians
Source: JMIR Serious Games. 2023 Apr 7;11:e43904. doi: 10.2196/43904 (PMC10131700; doi:10.2196/43904)
Supplement: Multimedia Appendix 2 [file games_v11i1e43904_app2.pdf]

Multimedia Appendix 2. Modifications made to bWell-D tasks after end-user feedback

	Suggested changes				
	Multisensorial / multidomain	Ecological	Challenging	Use at home /ease of use	Target mood / hot cognitions
**Tutorial and general interface**					
• Patient preferences menu allows user to choose between practice with a tutorial or performing the task				X	
• Clinical preferences menu for user to configure a given task (e.g., difficulty levels, presence of distractors)				X	
• Inclusion of bridging exercises post VR session: ask patients to reflect on how they think the tasks may apply to their personal context		X			
• VR program can be delivered on headsets that are tethered (ex: HTC Vive Pro) or standalone (e.g., Pico Neo 3 Eye, suited for use at home)				X	
**Egg**					
• Choice in environment: office vs park		X			
• Addition of realistic (office-related) targets		X			
• In game information displayed to user as a wrist menu (e.g., score, game mode, current target)				X	
• Ambient distractors:<br>○ Visual: Computer screens, co-workers, distractor eggs<br>○ Audio: Phone ring/people talking/buzzing sound<br>○ Co-workers engaging in distracting and emotionally charged conversation	X		X		X
• Pre-set variant of task: version where user follows auditory, rather than visually presented instructions	X				
• Adaptive difficulty progression modified to include visual, audio and tactile elements	X		X		
• Pre-set difficulty progression: simple to more complex real-world “mini-tasks” (e.g., find new target, go to target location, press button sequence to obtain bonus points)		X	X		
**Lab**					
• Supervisor present, observing and providing audio feedback on performance		X		X	X
• Supervisor can give distracting/non-relevant comments or negative feedback					X
• Addition of distractor beakers and target colours		X	X		
• Pre-set variant of task: version where user follows auditory, rather than visually presented instructions	X	X			
• New component in pre-set difficulty progression: integration of a secondary “mini” task where users must first mix colours to obtain required colour (mixing chart provided).		X	X		
**Mole**					
• Visual graphics signal score streak to user				X	
• Smiling / frowning moles, to test for bias for negative stimuli					X

• Pre-set variant of task: version designed to require additional physical effort (side moles and directional hits)	X		X		
• Pre-set variant of task: version that includes frustrating elements (multiple hits moles)					X
**Theater**					
• Choice in environment: theater vs classroom		X			
• Presence of teacher in front and audience members seated behind user		X			X
• Version of task where user follows auditory, rather than visual presentation	X				
• Inclusion of distractor objects (non-target shapes)			X		
• Instructor seated in view and writing notes, whispers as distraction					X
**Tent (additional activity, included from bWell platform core battery of exercises)**					
• Immersion in nature with self-guided exploration and duration. Breathing guide provided as an in-scene sphere that shrinks and grows.					X
• Choice in environment: the nature scene and accompanying music can be changed according to user preference by flipping through a catalogue.				X	
