# Supplementary material for: Co-design of a Virtual Reality Cognitive Remediation Program for Depression (bWell-D) With Patient End Users and Clinicians: Qualitative Interview Study Among Patients and Clinicians
Source: JMIR Serious Games. 2023 Apr 7;11:e43904. doi: 10.2196/43904 (PMC10131700; doi:10.2196/43904)
Supplement: Multimedia Appendix 3 [file games_v11i1e43904_app3.pdf]

Multimedia Appendix 3. Changes made to bWell-D after initial end-user feedback

General interface, tutorial, and bWell-D administration
• Tutorials were developed for each task in order to 1) provide opportunities for participants to practice and gain familiarity with VR and the controls, and 2) to ensure that participants with different baseline levels of familiarity with the technology were all still able to carry out the required VR interactions in each task.<br>• Clear menus that allow the customization of bWell-D, divided in 1) Patient preferences, which let the user decide on individual options (ex: select tutorial or perform task), and 2) Clinical preferences, which enables setting a given exercise to a particular configuration.<br>o Clinical preferences were grouped as: The choice between assessment and training mode, multidomain/multisensorial stimuli, hot cognition and pre-set difficulty levels that include distractors and rewards.<br>• Inclusion of 'bridging exercises' as a supplement to the VR program, which consist of asking participants to reflect on how they think the VR tasks will apply to their personal contexts to help them understand how practiced skills could be related to their real life.<br>• Based on the preference to have an option for an at-home intervention, bWell's hardware has an agnostic core that has now been leveraged to accommodate this option. This includes the integration of standalone headsets such as the Pico Neo 3 Eye and the Oculus Quest 2.
**Egg task**
• End-users recommended to include more ecologically valid VR elements. To address this, a more applicable virtual environment representing an office was created.<br>• The egg task environment was adapted to include new, more context-appropriate items for the office-themed virtual environment. These include staplers, legal pads and coffee mugs.<br>• To address the need for autonomy, an in-game user interface (UI) for the egg scene was added to the user's wrist to convey important information such as the current score, target object and target location.<br>• Ambient, multisensorial distractors were added as options to the scene. These distractors add artificial challenge, as well as addressing the mood theme by introducing an element of hot cognition.<br>o Visual distractors include videos playing on computer screens, co-workers walking around the office, and fake eggs that look almost like the real target eggs.<br>o Audio distractors include phone rings, coworkers talking, and a buzzing sound that emanate from random locations within the office.<br>o A customization option was added in which users follow auditory rather than the usual visual instructions.<br>• An adaptive difficulty progression including multisensorial stimuli was developed for the scene. To address the user's capability, difficulty adjusts based on user performance making this a personalization element.<br>o Difficulty adjustment includes adjusting the number of targets, the reaction delay allowed to get bonus points, the time it takes to hatch an egg and the number and frequency of audio distractors.<br>o The user's performance is assessed based on how they respond to multiple stimuli: Visual stimulus (egg turns green), tactile stimulus (controller vibrates) or audio stimulus (clock ticking sound clip).<br>o A pre-set difficulty progression was added to the scene, where participants start with simple tasks at lower levels and become increasingly complex at higher levels. These tasks include: finding a new target, going to a target location and pressing a button sequence to obtain bonus points.
**Lab task**
• A supervisor avatar is present in the scene and adds artificial assistance by giving auditory feedback on the user's performance and advice on how to improve.<br>o In order to add an aspect of hot cognition the avatar behaviour was modified so it can give distracting or irrelevant comments to the user.<br>o A customization option was added in which the supervisor avatar gives the user auditory instructions rather than having the recipes visually presented on tablets.<br>• To add artificial challenge, two modifications were made to the scene explicitly:<br>o The addition of three new not-relevant ingredient bottles, which distract the user while they attempt to complete their recipes.

    - The introduction of new secondary ingredients to recipes which are created by combining two primary ingredients in a beaker before pouring it in the tub, adding complexity to the task. A chart showing possible ingredient combinations was added to the scene.

Mole task

- A score streak animation was added as a gamification element (visual reward), aimed as level/progress feedback to motivate the user into keeping a fast pace to match the increasing difficulty. This occurs when multiple moles are correctly hit in sequence.
- The moles were modified to have a simple facial expression, either a smile or a frown. The program checks if the particular facial expression influences the accuracy of the hits.
    - In one mode, valid moles (ones with a color matching a hammer) are assigned a smiling expression and invalid moles are frowning.
    - In another mode, the expressions are randomized.
- A multiple hit mole (MHM) customization variant was added to target mood. MHM is a variant taken from the bWell core battery of exercises, designed to frustrate the user by requiring repeated hits in order for it to go down.
- Side moles were included to the side of the main table, requiring the user to turn their head and extend their arms further in order to hit them. This directional hit variant requires additional physical effort or dexterity.
    - Moles must be hit on the top of their head in order to score points. This is more challenging compared to the usual case in which moles can be hit from any angle and still score points.

Theater task

- This task was initially implemented with a theater-themed virtual environment. Later, a more ecologically valid classroom-themed environment was created, which now includes a teacher's desk in front, and the students' desks laid out in a half-circle in front of it.
    - The user is placed in front of the teacher who is seated at their desk. A few student avatars who may be enabled or disabled are seated in the classroom behind the user. This arrangement was intended to be somewhat daunting.
    - For additional distraction, the teacher in front can create noise by taking down notes and the student avatar scan make loud whispering noises. The arrangement and the distractions add a level of artificial challenge for the user as well as hot cognition.
- Further artificial challenge was added by including shapes that are not part of the correct answer.
- A customization option was added to the scene in which a virtual voice gives instructions about which shape to place in which order instead of presenting the target shapes visually.
    - Anticipating that this audio mode would be too difficult when the presented shapes were complex or unfamiliar a new collection of number-shaped objects was created for use in the auditory mode.

Tent scene

- While this scene was part of the bWell core platform, it was not initially included in bWell-D. Feedback from end-users about the need for activities that target mood prompted its inclusion.
- The user is placed in a relaxing nature environment and allowed to freely explore.
- A ball that grows and shrinks to guide the user's breathing is included, as well as additional user customization promoting relaxation.
- The user is given control of the selection of the environment and background music that plays.
    - A virtual book placed in front of the user includes controls which allow them to navigate through a variety of virtual environments.
- The scene was designed to follow an exploratory/open-world approach, allowing users to freely look around the environment for as much or as little time as they wish.
