# Supplementary material for: Co-design of a Virtual Reality Cognitive Remediation Program for Depression (bWell-D) With Patient End Users and Clinicians: Qualitative Interview Study Among Patients and Clinicians
Source: JMIR Serious Games. 2023 Apr 7;11:e43904. doi: 10.2196/43904 (PMC10131700; doi:10.2196/43904)
Supplement: Multimedia Appendix 5 [file games_v11i1e43904_app5.pdf]

**Multimedia appendix 5.** Qualitative interview script, bWell-D (re-interview)

*Length: 45 minutes*

1. **Primary goal**

In the last interview, we showed you some video clips from bWell-D, a virtual reality program designed to help improve cognitive symptoms associated with depression (problems with memory, attention, multi-talking). Based on the feedback that you and other participants provided, we have modified bWell-D. We would now like to show you the updates we have made to the program, so you can let us know whether we have attended to your suggestions appropriately.

2. **Verbal consent**

- Would you like to participate in this interview?
- Do you agree to be recorded?

3. **Description of the session**

Firstly, I'll provide you some background information about how bWell-D works for enhancing cognition *[psychoeducation]*. Then, I'll briefly remind you what each task is about, I'll show you the video you saw on our previous interview, followed by its updated version, and I'll ask you some questions afterwards.

4. **Psychoeducation section**

bWell-D is a VR cognitive remediation treatment for depression. Cognitive remediation is a type of treatment that aims to improve several cognitive domains that often get affected when someone is depressed (e.g., attention, memory, and problem solving). This is meant to be achieved through the repeated practice of skills and exercises, which often involve playing memory or concentration 'games' on a computer, and they become harder as you progress. Some research suggests that the tasks you practice in a cognitive remediation treatment could enhance your performance in your day-to-day life (e.g., at work, home, or school).

bWell-D is formed by the four main tasks I showed you on our last interview: Egg, Lab, Mole and Theater. These tasks are designed to operate in three modes: **1) tutorial**, **2) assessment**, and **3) training. Tutorial** has the aim of familiarising the patient with the virtual environment, and to show them the actions they will have to perform in the tasks. Then comes the **assessment** mode, which has the purpose of identifying patients' individual needs by assessing their task performance, with a fixed difficulty level. In the following

mode, **training**, the level of difficulty is adaptable based on the patients' performance, so it is never too easy or too hard. In training mode, real-time feedback is provided to the patient, showing them level or score changes, as well as successes and errors.

5. **bWell-D videos description, original and updated versions, and follow-up questions**

In this following part of the interview, I will show you the old and updated videos of bWell-D, followed by some questions. For this, I will share my computer screen and sound with you.

**<u>General interface</u>.** Firstly, I would like to show you the menu and all the settings that you can modify in bWell, so the session gets personalized according to the patients' needs.

*[Show UI videos]*

- Show options in clinical preferences menu (assessment vs training, multidomain, multisensorial)
- Show customisation that target 'hot cognition' (cognitive responses that can be affected by mood)
- Show multiple segments showing the different levels of difficulty
- Show score
- Show distractors

- What do you think of this menu? Any changes you would suggest?

a) **Egg task.** In this task, participants are asked to look around and find eggs in an office environment. They can walk around or use 'teleporters' to explore the space. When they find an egg, they have to fix their gaze on the egg until it pops. If they break their gaze, the egg does not pop. This task is meant to target sustained attention.

*[Show original video]*

*[Show updated version]*

**Follow-up questions for Egg task**

- What main differences did you notice between the two versions of this task?

<u>(If not elicited) New elements:</u>

- Choice in environment: Office vs park
- Multisensorial bonus options: Audio/visual/tactile or random
- Ambient distractors:
  - Visual: Computer screens, wanderers, distractor eggs
  - Audio: Phone ring/chatter/mosquito

        - ‘Hot cognition’ = wanderers saying “hello”
    - Adaptive difficulty progression rises
        - Start level of difficulty = assessment: eggs with equal size, hatching time and value, distractor eggs (stripe), infrequent audio
        - Medium level of difficulty = training: eggs with variable size, hatching time and value, distractor eggs (stripe), infrequent audio
        - Hard level of difficulty = training as above, distractor eggs (shape), frequent audio, long hatch, short bonus window, eggs decrease in value faster
- What do you think of these modifications?
- Which version of the task do you prefer?
- Would you modify anything else?
- *[Mention potential future developments and obtain participant feedback]*
    - Include more complex verbal interactions:
        - Have verbal directions come from people in the office
        - Distracting co-workers giving non-relevant instructions/chatter
        - Have co-workers who require tasks to be completed urgently
    - Simple verbal instructions:
        - “Please try to find the blue eggs, striped eggs, etc.”
        - “Go straight through the hallway, turn left, go the kitchen area/boardroom, etc.” OR towards a specific teleport
    - Verbal directions become more complex
        - “When you get to kitchen, only pick out the red eggs, in board room only pick out the blue ones”
    - Incorporate small ‘tasks’:
        - “Go towards the red teleport, and collect two red eggs and one blue egg”, then receive rewarding stimuli”
    - Have a greater density of visual distractors (people/computer screen)
- [If not addressed in previous questions]: How well do you think the updated version targets cognitive difficulties you may have experienced?
- How do you think this task may apply to your personal context (at home/ at work / at school)? Are there specific tasks in your day-to-day life that could benefit from this kind of practice of sustained attention?

b) **Lab task.** In this task, participants are asked to follow instructions for two recipes in a lab simultaneously. The participant has to follow the instructions showed

to them on two tablets placed nearby. Participants have to go back and forth between recipes. This task is meant to target multitasking and divided attention.

*[Show original video]*

*[Show updated version]*

**Follow-up questions for Lab task**

- What main differences did you notice between the two versions of this task?
  <u>(If not elicited) New elements:</u>
  - Supervisor present, provides feedback
  - Score and timeline on tablet for feedback on performance
  - ShowEndAnimations (fireworks) gamification element
  - Multisensorial = gaze and controller interactions (grab, pour and release)
  - 'Hot cognition' = show avatar (observing performance)
  - Difficulty levels:
    - Start level= Assessment: Tablet position is fixed, recipes start automatically, no avatar, fixed criteria for success (80%)
    - Medium level= Training: Show score, user can position the tablet and must start each recipe, avatar providing feedback
    - Hard level= Training as above, recipes with higher number of steps, longer duration, smaller pour window
- What do you think of these modifications?
- Which version of the task do you prefer?
- Would you modify anything else?
- [Mention potential future developments and obtain participant feedback]
  - Incorporate audio elements:
    - E.g., Instead of visual guide, can include a mode with verbal instruction (main goal is auditory) at later stages of training
    - E.g., Can give distracting/non-relevant instructions/conversations
  - Can include negative feedback from supervisor
  - Include more distractor beakers
  - Increase number of target colours
- [If not addressed in previous questions]: How well do you think the updated version targets cognitive difficulties you may have experienced?
- How do you think this task may apply to your personal context (at home/ at work / at school)? Are there specific tasks in your day-to-day life that could benefit from this kind of practice of sustained attention?

c) **Mole task.** This task is similar to the ‘whack-a-mole’ game. In this task, participants are asked to hit shapes as they pop up from the table with one of the hammers that they are holding. The participant has to hit the shape with a hammer that is a matching color. The color of the hammers and the shapes change throughout the game. This task is meant to improve how quickly a person reacts, and their ability to stop themselves from reacting when appropriate.

*[Show original video]*

*[Show updated version]*

**Follow-up questions for Mole task**

- What main differences did you notice between the two versions of this task?
  <u>(If not elicited) New elements:</u>
    - Score streak for gamification element
    - Multisensorial: Audio and tactile (vibration in controllers) feedback
    - ‘Hot cognition’ = Smiling moles
    - Test for bias with smile for valid moles vs invalid moles
    - Difficulty levels:
        - Start level = Assessment: Moles fixed in terms of number, time shown and interval between moles popping-up
        - Medium level = Training: Show score, adaptive difficulty, smiling moles
        - Hard level = As above with increasing number of moles and decrease in show time, interval between one mole appearing after another
- What do you think of these modifications?
- Which version of the task do you prefer?
- Would you modify anything else?
- *[Mention potential future developments and obtain participant feedback]*
    - More colours in the mole task
    - Having a few more ‘distractor’ colours amongst the moles (that never matches the hammers)
- [If not addressed in previous questions]: How well do you think the updated version targets cognitive difficulties you may have experienced?
- How do you think this task may apply to your personal context (at home/ at work / at school)? Are there specific tasks in your day-to-day life that could benefit from this kind of practice of sustained attention?

d) **Theatre task.** In this task, participants are shown a sequence of shapes for a short period of time. They are then asked to recreate the sequence by selecting the correct shapes from a pool of objects and placing these shapes in the correct order. This task is meant to target memory

*[Show original video]*

*[Show updated version]*

**Follow-up questions for Theatre task**

- What main differences did you notice between the two versions of this task?
  <u>(If not elicited) New elements:</u>
  - Audience members seated behind
  - Choice in environment: Theater vs classroom
  - Distractors = artefact type: #objects in easy, original and abstract = 6, 12 and 12 respectively (larger sets have more non-target shapes)
  - ‘Hot cognition’ = Instructor seated in view and writing notes, whispers as distraction
  - Difficulty levels
    - Start level= Assessment: Fixed view time (encoding), start after all artefacts have fallen (retention) and duration (retrieval)
    - Medium level = Training: Can start as soon as one object falls and adaptive levels of difficulty, small number of artefacts, easy artefacts
    - Hard level = Training as above with higher number of artefacts and abstract artefacts
- What do you think of these modifications?
- Which version of the task do you prefer?
- Would you modify anything else?
- *[Mention potential future developments and obtain participant feedback]*
  - Left (yellow) button provides a hint
  - Add audio component to theatre task:
    - E.g., memorizing verbally expressed words, remembering the order of complex instructions
    - E.g., This could be a separate task to run in addition to the theatre task (dual task)
  - Add other types of stimuli, e.g., words/numbers
  - Can increase number of distractor objects
  - Have a larger pool of objects that continually change over the course of training

- [If not addressed in previous questions]: How well do you think the updated version targets cognitive difficulties you may have experienced?
- How do you hink this task may apply to your personal context (at home/ at work / at school)? Are there specific tasks in your day to day life that could benefit from this kind of practice of sustained attention?

e) **Tent (relaxation).** This is a new task that you didn't see in our previous interview. This is a bWell-D activity that has the purpose of placing the participant in a relaxing environment. It displays a tent in a sunset scenery, accompanied by relaxing music. Inside the tent, there is a ball that shrinks and grows, and it's intended to function as a breathing guide. The outside environment can be changed by flipping the page of a book placed nearby and touching the selected environment.

*[Show video]*

**Tent follow-up questions**

- What do you think of this activity?
- Would you modify anything in this task?
- [If not addressed in previous questions]: How well do you think the activity might help you relax?
- Could you see yourself using the Tent activity? Is this something that could be useful for you to practice? Why or why not?

**6. General questions**

a) Several participants expressed they would like to carry out the treatment at home. The treatment is intended to follow a hybrid model, including an in-person assessment session, and continuing with the VR treatment at home. What do you think of this treatment format?

b) Some cognitive remediation programs using 'bridging exercises'. This is where a therapist will sit down with the individual to discuss the tasks they have completed, strategies they used while completing the tasks, and specific situations in their life where they could implement these strategies. Would this be helpful to you if you were to complete the bWell-D training program? Why or why not?

c) Are there any other types of tasks you would suggest?

d) Do you expect any technical issues getting in the way when doing the treatment from home?
