# Supplementary material for: Co-design of a Virtual Reality Cognitive Remediation Program for Depression (bWell-D) With Patient End Users and Clinicians: Qualitative Interview Study Among Patients and Clinicians
Source: JMIR Serious Games. 2023 Apr 7;11:e43904. doi: 10.2196/43904 (PMC10131700; doi:10.2196/43904)
Supplement: Multimedia Appendix 1 [file games_v11i1e43904_app1.pdf]

Patients' qualitative interview script

Qualitative interview introduction

Length: 45 minutes

Primary goal: To obtain your perspective and thoughts about virtual reality in clinical care, and its potential use in a cognitive remediation intervention

1) **Verbal consent**

   Would you like to participate in this interview?

   Verbal consent was **obtained** from the participant

   Verbal consent was **NOT obtained** from the study participant

   Do you agree to be recorded?

2) **Personal experience with virtual reality**

   Virtual reality is a computer generated 3-D simulation of an environment that the user can interact with (often by wearing a headset and having special controllers).

   - Do you have any experience using virtual reality in your personal life?
   - Where experienced
   - Nature of experience (demo, gaming)
   - Was your experience positive or negative? Why?

   If no experience: Is it something that you are interested in trying? Why or why not?

3) **Clinical experience with virtual reality**

   - Have you ever received any type of psychiatric/psychological treatment through virtual reality?
     - If unclear, can elaborate: for example, some people with anxiety receive exposure therapy through virtual reality, or do mindfulness meditation exercises in virtual reality
   - To what purpose was the VR used?

- Could you describe the type of VR program you used *[information to elicit: Immersive vs non-immersive, duration/frequency, type of tasks/games]*
- Did you consider this VR intervention helpful? Why / why not?
- Were there any challenges or difficulties while receiving the VR treatment?

<u>If no clinical experience with virtual reality:</u> Have you heard of VR being used <u>to treat psychiatric disorders</u>

- If yes: For what purposes?
- If no: Are you interested on learning about VR in clinical settings? Why / why not?

4) Perceptions about virtual reality in clinical settings

- Given your experience or what you know about VR, would you be open to trying a VR therapy for your psychiatric disorder? Why or why not?
- For which purposes do you think VR could be used?
  - <u>If not elicited:</u> How relevant do you think a VR intervention would be for patients specifically with depression?
- What barriers do you see for the use of VR in clinical settings?
- What factors might encourage people to undergo a VR treatment for mental health?

5) Goals for cognitive/functional remediation

- Have you faced any challenges in your job/school related to your depression diagnosis?

Now I am going to ask some questions about cognitive remediation. Cognitive remediation is a behavioral based training method that aims to improve cognition (e.g. memory, attention and problem solving) through the repeated practice of skills and exercises.

- Have you ever undertaken a cognitive remediation treatment?
- Have you undertaken a cognitive remediation treatment since your depression diagnosis?

If yes: Describe the type of cognitive remediation (specific programs used, duration, frequency)

If no: Are you familiar with the use of cognitive remediation in psychiatric populations?

- Is it something you would be interested in trying as part of your depression treatment? Why / why not?
- What specific outcomes would you like to see in yourself as a result of a cognitive remediation program for your depression?

6) Remote work changes due to Covid-19

- Have you been using any virtual/technological tools (video conferencing, computerized tasks, etc) since March 2020?

  If yes
- How do you feel about receiving therapy this way?
- Have you found any benefits in utilising these sorts of virtual/technological tools?
- Have there been any issues?

  If no:
- Why not? [Information to elicit: Patient dropped out or clinician stopped treatment]
- Were you aware of virtual tools for continuing mental health treatments?

- Has your opinion on using virtual tools and/or virtual reality changed as a result of your experiences due to covid-19? Why or why not?
- If you were offered a VR cognitive remediation treatment, would you be interested in trying it?

  If yes: Would you like to try it at a clinic or at home? Why?

  If no: Why not? What could motivate you to try it?

7) Videos about tasks from a cognitive remediation program through VR.

Get the patients' opinion about the videos, ask whether they consider the videos target the intended cognitive domain appropriately, and get any suggestions for improvement.

We are now going to show you video clips from a virtual reality cognitive remediation program for people with depression. In this program, individuals would complete each task

for 10 minutes, twice a week for 8 weeks. The tasks get progressively harder over each session. Individuals would be wearing a headset and have controllers to interact with the environment. We will describe the task, show the video and then ask you some questions afterwards.

a. <u>Egg task (video 1a office)</u>

   Description: In this task, participants are asked to look around and find eggs in an office environment. They can walk around or use 'teleporters' to explore the space. When they find an egg, they have to fix their gaze on the egg until it hatches. If they break their gaze, the egg does not hatch.

   Questions after video: What specific skills do you think this task is meant to develop?
   [If not elicited, explain that this task is meant to target sustained attention]
   How well do you think this task targets sustained attention?
   Do you have any suggestions for improving or modifying the task?

b. <u>Lab task (video 2 avatar)</u>

   Aim: In this task, participants are asked to follow instructions for two recipes in a lab simultaneously. The participant has to follow the instructions showed to them on two tablets placed nearby. Participants have to go back and forth between recipes.

   Questions after video: What specific skills do you think this task is meant to develop?
   [If not elicited, explain that this task is meant to improve dividing attention between tasks and multitasking]
   How well do you think this task targets divided attention and multitasking?
   Do you have any suggestions for improving or modifying the task?

c. Mole task (video 3a moles as cylinders)

   Aim: This task is similar to the 'whack-a-mole' game. In this task, participants are asked to hit shapes as they pop up from the table with one of the hammers that they are holding. The participant has to hit the shape with a hammer that is

a matching color. The color of the hammers and the shapes change throughout the game.

Questions after video: What specific skills do you think this task is meant to develop?
[If not elicited, explain that this task is meant to target reaction time and inhibiting response to incorrect stimuli]
How well do you think this task targets reaction time and response inhibition?
Do you have any suggestions for improving or modifying the task?

d. Theatre task (video 4b classroom environment)
Aim: In this task, participants are shown a sequence of shapes for a short period of time. They are then asked to recreate the sequence by selecting the correct shapes from a pool of objects, and placing these shapes in the correct order.

Questions after video: What specific skills do you think this task is meant to develop?
[If not elicited, explain that this task is meant to target memory]
How well do you think this task targets memory?
Do you have any suggestions for improving or modifying the task?

- Would you like to participate in a treatment that include these tasks? Why / why not?
- Do you foresee any obstacles when undertaking this treatment?
- Is there anything you think would be helpful to add to this program?

Clinicians' qualitative interview script

Qualitative interview introduction

Length: 30-45 minutes

Primary goal: To obtain your perspective and thoughts about virtual reality in clinical care, and virtual reality for cognitive remediation

8) Verbal consent

Would you like to participate in this interview?

Verbal consent was obtained from the participant

Verbal consent was NOT obtained from the study participant

Do you agree to be recorded?

9) Personal experience with virtual reality

Virtual reality is a computer generated 3-D simulation of an environment, that the user can interact with (often by wearing a headset and having special controllers).

Do you have any experience using virtual reality in your personal life?

    Where experienced

    Nature of experience (demo, gaming)

    Was your experience positive or negative? Why?

    <u>If no experience</u>, is it something that you are interested in trying? Why or why not?

10) Clinical experience with virtual reality

Have you ever used virtual reality for a clinical application?

    To what purpose was the VR used?

    Could you describe the type of VR program you used [information to elicit: immersive vs non-immersive, duration/frequency, type of tasks/games]

    In what patient population was the VR used?

    Perceptions of success of VR intervention

What feedback have you received from patients regarding VR?

Challenges in using VR

If no clinical experience with virtual reality: Have you heard of VR being used in clinical settings? If yes: for what purposes?

If no: Are you interested on learning about VR in clinical settings? Why / why not?

11) Perceptions about virtual reality in clinical settings

Given your experience or what you know about VR, do you think VR interventions are relevant in clinical settings with psychiatric populations?

Why or why not?

Purposes for which VR could be used

Patient populations for which VR might be helpful

If not elicited: How relevant would a VR intervention be for patients specifically with depression?

What barriers do you see for the use of VR in clinical settings?

What factors might encourage you to use VR in clinical settings?

12) Goals for cognitive/functional remediation

What are the common challenges that patients with depression encounter in their jobs/school or when trying to return to work/school?

Now I am going to ask some questions about cognitive remediation.

Cognitive remediation is a behavioral based training method that aims to improve cognition (e.g. memory, attention and problem solving) through the repeated practice of skills and exercises.

Have you ever implemented cognitive remediation with your patients?

If yes: Describe the type of cognitive remediation (specific programs used, duration, frequency)

How routinely do you implement cognitive remediation with patients?

For which types of patients do you use cognitive remediation?

Have you ever used cognitive remediation with patients with depression?

If yes: what was your experience? Perceptions of success/barriers

If no: Would you consider implementing for patients with depression? Why or why not?

If no: Are you familiar with the use of cognitive remediation in psychiatric populations?

If not elicited: Are you familiar with the use of cognitive remediation in depression specifically?

Is it something you would consider implementing for patients with depression? Why or why not?

What specific patient outcomes would you want to see as a result of a cognitive or functional remediation program for patients with depression?

If you were going to implement a cognitive remediation program through VR for patients with depression, what type of information would it be helpful for you to obtain to monitor how the patient is doing with the program?

13) Remote work changes due to Covid-19

Have you been using any virtual/technological tools (video conferencing, computerized tasks, etc) since March 2020?

How do you feel about delivering therapy this way?

How did your patients respond to this new treatment modality?

Have you found any benefits in utilising these sorts of virtual/technological tools?

Have there been any issues?

Where did you learn about the virtual/technologocal tools you are using?

<u>If no:</u>

Why not?

How have you managed to continue treating your patients?

Were you aware of virtual tools for continuing treatment with patients?

Has your opinion on using virtual tools and/or virtual reality changed as a result of your experiences due to covid-19? Why or why not?

Would a virtual reality program in ourclinical setting be feasible?
Why or why not?
Would a virtual reality program that patients can use at home be feasible?
Why or why not?

14) Videos about tasks from a cognitive remediation program through VR. *Get the clinicians' opinion about the videos, ask whether they consider the videos target the intended cognitive domain appropriately, and get any suggestions for improvement.*

We are now going to show you video clips from a virtual reality cognitive remediation program for people with depression. In this program, patients would complete each task for 10 minutes, twice a week for 8 weeks. The tasks get progressively harder over each session. Patients would be wearing a headset and have controllers to interact with the environment. We will describe the task, show the video and then ask you some questions afterwards.

a. Egg task (video 1a office)
   Description: In this task, participants are asked to look around and find eggs in an office environment. They can walk around or use 'teleporters' to explore the space. When they find an egg, they have to fix their gaze on the egg until it hatches. If they break their gaze, the egg does not hatch.

   Questions after video: What specific skills do you think this task is meant to develop?
   [If not elicited, explain that this task is meant to target sustained attention] How well do you think this task targets sustained attention?
   Do you have any suggestions for improving or modifying the task?

b. Lab task (video 2 avatar)

Aim: In this task, participants are asked to follow instructions for two recipes in a lab simultaneously. The participant has to follow the instructions showed to them on two tablets placed nearby. Participants have to go back and forth between recipes.

Questions after video: What specific skills do you think this task is meant to develop?
[If not elicited, explain that this task is meant to improve dividing attention between tasks and multitasking] How well do you think this task targets divided attention and multitasking?
Do you have any suggestions for improving or modifying the task?

c. Mole task (video 3a moles as cylinders)
Aim: This task is similar to the 'whack-a-mole' game. In this task, participants are asked to hit moles as they pop up from the table with one of the hammers that they are holding. The participant has to hit the mole with a hammer that is a matching color. The color of the hammers and the moles change throughout the game.

Questions after video: What specific skills do you think this task is meant to develop?
[If not elicited, explain that this task is meant to target reaction time and inhibiting response to incorrect stimuli] How well do you think this task targets reaction time and response inhibition?
Do you have any suggestions for improving or modifying the task?

d. Theatre task (video 4b classroom environment)
Aim: In this task, participants are shown a sequence of shapes for a short period of time. They are then asked to recreate the sequence by selecting the correct shapes from a pool of objects, and placing these shapes in the correct order.

Questions after video: What specific skills do you think this task is meant to develop?

[If not elicited, explain that this task is meant to target memory] How well do you think this task targets memory?
Do you have any suggestions for improving or modifying the task?

- How do you think your patients might respond to these tasks?

- What barriers do you think would come up when utilising this program with your patients?

- Is there anything you thinkwould be helpful to add to this program?
